# A zebrafish model of combined saposin deficiency identifies acid sphingomyelinase as a potential therapeutic target

**DOI:** 10.1242/dmm.049995

**Published:** 2023-06-27

**Authors:** Tejia Zhang, Ivy Alonzo, Chris Stubben, Yijie Geng, Chelsea Herdman, Nancy Chandler, Kim P. Doane, Brock R. Pluimer, Sunia A. Trauger, Randall T. Peterson

**Affiliations:** ^1^ Department of Pharmacology and Toxicology, University of Utah, Salt Lake City, UT 84112, USA; ^2^ Bioinformatic Analysis Shared Resource, Huntsman Cancer Institute, University of Utah, Salt Lake City, UT 84112, USA; ^3^ Department of Neurobiology and Molecular Medicine Program, University of Utah School of Medicine, Salt Lake City, UT 84112, USA; ^4^ Electron Microscopy Core Laboratory, University of Utah, Salt Lake City, UT 84112, USA; ^5^ Harvard Center for Mass Spectrometry, Harvard University, Cambridge, MA 02138, USA

**Keywords:** Lipidomics, Lysosomal storage disease, Zebrafish

## Abstract

Sphingolipidoses are a subcategory of lysosomal storage diseases (LSDs) caused by mutations in enzymes of the sphingolipid catabolic pathway. Like many LSDs, neurological involvement in sphingolipidoses leads to early mortality with limited treatment options. Given the role of myelin loss as a major contributor toward LSD-associated neurodegeneration, we investigated the pathways contributing to demyelination in a CRISPR-Cas9-generated zebrafish model of combined saposin (*psap*) deficiency. *psap* knockout (KO) zebrafish recapitulated major LSD pathologies, including reduced lifespan, reduced lipid storage, impaired locomotion and severe myelin loss; loss of *myelin basic protein a* (*mbpa*) mRNA was progressive, with no changes in additional markers of oligodendrocyte differentiation. Brain transcriptomics revealed dysregulated mTORC1 signaling and elevated neuroinflammation, where increased proinflammatory cytokine expression preceded and mTORC1 signaling changes followed *mbpa* loss. We examined pharmacological and genetic rescue strategies via water tank administration of the multiple sclerosis drug monomethylfumarate (MMF), and crossing the *psap* KO line into an acid sphingomyelinase (*smpd1*) deficiency model. *smpd1* mutagenesis, but not MMF treatment, prolonged lifespan in *psap* KO zebrafish, highlighting the modulation of acid sphingomyelinase activity as a potential path toward sphingolipidosis treatment.

## Teaser

**Editors' choice:** Using a new zebrafish model of combined saposin deficiency, a lysosomal storage disease, we examine both pharmacological and genetic approaches toward sphingolipidosis treatment.

## INTRODUCTION

Lysosomal storage diseases (LSDs) are a family of ∼70 metabolic disorders caused primarily by mutations in enzymes involved in lysosomal catabolism (Platt et al., 2018). Loss of enzyme activity results in pervasive substrate accumulation and multiple-organ pathologies that frequently involve neurodegeneration, which contributes to early death in a significant number of patients (Platt et al., 2018). The majority of known LSDs lack treatment options (Platt et al., 2018).

One approach toward LSD classification is based on substrate type. Under this system, sphingolipidoses constitute a prominent LSD class that affects the sphingolipid metabolic pathway (Özkara, 2004). Sphingolipids are a major lipid family with crucial functions in nearly all aspects of cell biology (Zhang and Saghatelian, 2013; Saddoughi and Ogretmen, 2013; Stancevic and Kolesnick, 2010). Sphingolipid metabolism is highly conserved across species, and perturbations in sphingolipid levels have been implicated in embryogenesis defects and metabolic syndromes (Mendelson et al., 2017; Sokolowska and Blachnio-Zabielska, 2019). Given the essentiality of sphingolipids for cellular function, loss-of-function mutations in virtually all enzymes of sphingolipid catabolism lead to sphingolipid accumulation and LSD pathologies (Özkara, 2004).

Like many other LSDs, major pathologies of sphingolipidoses include neurodegeneration, hepatosplenomegaly and impaired locomotion (Platt et al., 2018). Although common features exist among most sphingolipidoses, variations in clinical presentations across tissue types have also led to the development of sphingolipidosis subtype-specific diagnostic criteria (Platt et al., 2018). Similar to many LSDs, neurodegeneration is frequently involved in early-onset sphingolipidosis subtypes and leads to mortality within the first few years of life in the absence of treatment (Platt et al., 2018). Importantly, myelin loss is a major hallmark of the neuropathy associated with sphingolipidoses and additional LSDs, and can involve either or both the central nervous system (CNS) and the peripheral nervous system (Strölin et al., 2017; Biegstraaten et al., 2010; Suzuki, 2003).

Although enzyme replacement and small-molecule-based therapies have shown efficacy in the treatment of non-neuropathic forms of Gaucher disease (Ficicioglu, 2008; Peterschmitt et al., 2019; Andersson et al., 2005), treatment of neuropathic sphingolipidoses remains challenging owing to inefficient delivery of therapeutic agents across the blood-brain barrier (Santos and Amaral, 2019). To address this unmet need, more in-depth understanding of the mechanisms underlying disease progression, especially in the context of demyelination, could prove crucial in uncovering novel disease-associated pathways and drug targets. In recent years, the zebrafish has gained significant traction as a model organism for disease modeling (Seth et al., 2013). In addition to the amenability to genome editing and conservation of major organs and pathways, the zebrafish confers unique advantages including small size and optical transparency in the embryo/larva stage, which enable high-throughput applications that are otherwise challenging in rodents (Zhang and Peterson, 2020; Rennekamp and Peterson, 2015; Peterson et al., 2000). To date, three sphingolipidoses [Gaucher disease (Keatinge et al., 2015; Zancan et al., 2015; Lelieveld et al., 2019), Sandhoff disease (Kuil et al., 2019) and Farber lipogranulomatosis (Zhang et al., 2019)] have been modeled in the zebrafish, with good replication of human symptomologies. In addition to existing knockout models, the availability of CRISPR-Cas9-targeted mutagenesis strategies also allows the modeling of specific LSD-associated mutations in this organism (Liu et al., 2019; Komor et al., 2016; Anzalone et al., 2019). Importantly, the amenability of zebrafish larvae toward high-throughput chemical screening presents an invaluable avenue for the translation of model-based knowledge into drug discovery efforts (Peterson et al., 2000).

To expand the current repertoire of sphingolipidosis models and identify disease-relevant pathways translatable to therapy, we generated a zebrafish model of combined saposin deficiency using CRISPR-Cas9. Saposins A, B, C and D (all encoded by *psap*) are small glycoproteins that enhance the functions of various lysosomal sphingolipid catabolic enzymes (Kishimoto et al., 1992). As six of the 11 enzymes within the sphingolipid catabolic pathway function alongside at least one saposin, loss of function in one or multiple saposins leads to sphingolipidosis pathologies (Özkara, 2004). *psap* knockout (KO) zebrafish exhibited shortened lifespan, impaired locomotion and marked demyelination across the entire brain. Progressive *mbpa* loss was detectable starting from ∼1 month post fertilization (mpf), which was preceded by an overactivated neuroinflammatory response, but not by perturbations in mTORC1 signaling. Importantly, crossing the *psap* KO line with a zebrafish model of acid sphingomyelinase (*smpd1*) deficiency improved survival, supporting further exploration of acid sphingomyelinase modulation for sphingolipidosis treatment.

## RESULTS

### A zebrafish model of combined saposin deficiency

**Fig. 1.**
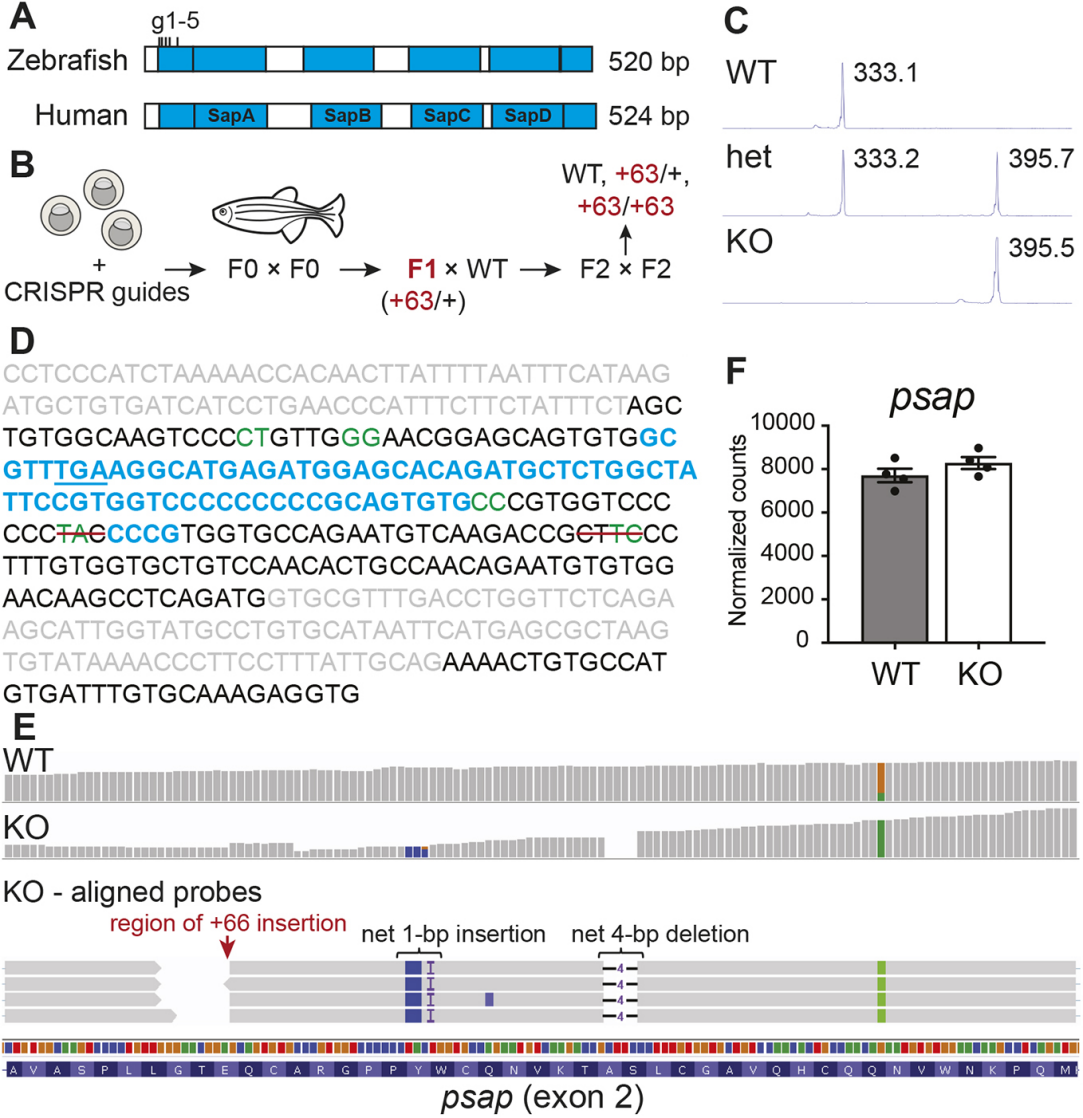
**A zebrafish model of combined saposin deficiency.** (A) Comparison between human (UniProt: P07602) and zebrafish (UniProt: B8JI17) saposin (blue) domains (detailed amino acid sequence alignment can be found in Fig. S1). CRISPR-Cas9 guide sites (located within exon 2) are indicated. (B) Schematic of combined saposin deficiency model generation in the zebrafish. (C) DNA fragment analyses of *psap* PCR fragments from WT and mutant zebrafish, demonstrating the presence of a net 63-bp insertion in the mutant sample. (D) Summary of Sanger sequencing results. Exon sequences are in black, intron sequences are in gray, predicted Cas9 cut sites are in green, insertions are in blue, and red lines indicate deletions. The in-frame stop codon (TGA) generated within the largest insertion sequence is underlined. Detailed Sanger sequencing data are in Fig. S1. (E) RNA sequencing probe alignments around the Cas9-targeted region in 4 mpf *psap^+63/+63^* and WT sibling brains, demonstrating preservation of the mutations detected at the genomic DNA level. (F) *psap* mRNA is not degraded based on RNA sequencing of 4 mpf *psap^+63/+63^* (*n*=4) and WT (*n*=4) sibling brains. Data show the mean±s.e.m.

Saposins A, B, C and D (SapA-SapD) are small glycoproteins formed from post-translational cleavage of the 58 kDa multifunctional precursor protein prosaposin (PSAP) (Leonova et al., 1996). Fig. 1A and Fig. S1 illustrate the known saposin domains (in blue) for human PSAP (UniProt: P07602) and zebrafish Psap (UniProt: B8JI17) (UniProt Consortium, 2018), demonstrating the presence of similar saposin domains in both systems. The amino acid sequence identities between the human and zebrafish proteins are 52%, 53%, 41%, 53% and 47% for SapA, SapB, SapC, SapD and PSAP, respectively (UniProt Consortium, 2018; Altschul et al., 1990).

To evaluate the impact of saposin loss in a vertebrate system, we used CRISPR-Cas9 to generate a zebrafish model of combined saposin deficiency. Five RNA guides were designed to target exon 2 of zebrafish *psap*, prior to the region encoding the four largest saposin domains corresponding to human SapA-SapD (Fig. 1A). A schematic of the model generation process is shown in Fig. 1B. Following guide delivery at the one-cell stage, the injected embryos were raised to adulthood and incrossed to yield F1 zebrafish, from which a fish carrying a heterozygous net 63-bp insertion in the *psap* locus was identified via DNA fragment analysis (Fig. 1C) and further verified by Sanger sequencing (Fig. S2A,C). The F1 fish of interest was outcrossed to yield heterozygous F2 offspring, which were then incrossed to yield F3 wild-type (WT), heterozygous and homozygous populations (Fig. 1B). A second F1 fish carrying a heterozygous net 14-bp deletion (Fig. S2B,C) was also identified and propagated to homozygosity following the same protocol.

Sanger sequencing of the *psap^+63/+^* F1 fish revealed the 63-bp insertion to be a combination of several mutations (Fig. 1D; Fig. S2A) at different predicted Cas9 cut sites, a consequence of the co-injection of multiple RNA guides. Together, the detected mutations translate to two missense mutations corresponding to amino acids 24 and 25, followed by a premature stop codon corresponding to amino acid 26 (early exon 2) of Psap, preceding the four longest saposin domains (Fig. 1A,D). These mutations were also preserved at the transcript level, as confirmed by RNA sequencing of WT and *psap^+63/+63^* adult zebrafish brains (Fig. 1E). *psap* mRNA expression did not vary significantly between *psap^+63/+63^* fish and WT siblings (Fig. 1F), suggesting that the mutations in the *psap* transcript failed to trigger nonsense-mediated mRNA decay. Notably, *psap* mutagenesis also led to the upregulation of additional genes of lysosomal sphingolipid catabolism (Fig. S3), likely as compensatory mechanisms to counteract potential sphingolipid storage in the absence of saposins.

### *Psap* KO zebrafish exhibit altered lipid metabolism

**Fig. 2.**
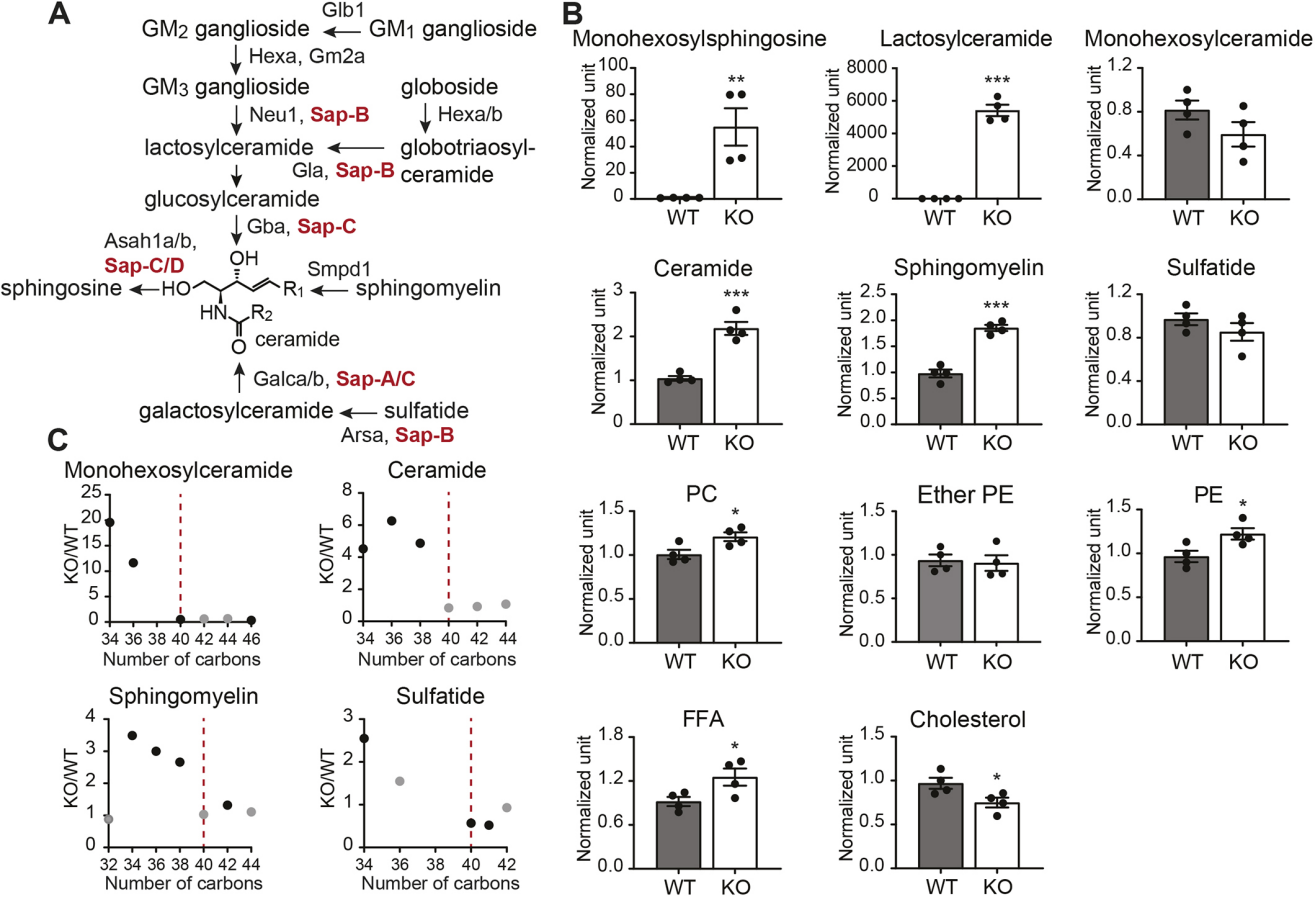
***Psap* KO zebrafish exhibit altered lipid metabolism.** (A) The sphingolipid catabolic pathway. The illustration is based on Özkara (2004). (B) Relative levels of major lipid families in 4 mpf WT (*n*=4) and *psap^+63/+63^* (*n*=4) brains. PC, phosphatidylcholine; PE, phosphatidylethanolamine; FFA, free fatty acid. (C) KO/WT fold changes versus the number of carbons in the ceramide core of the lipid species for selected sphingolipid families, demonstrating a rapid drop in the amplitude of lipid accumulation at around 40 carbon atoms, corresponding to a C22 acyl chain for the majority of sphingolipids given the prevalence of the d18 backbone in adult zebrafish brains (Zhang et al., 2019). Data points in black are statistically significant. Additional lipidomics data are in Fig. S3 (4 mpf WT and *psap^−14/−14^* brains) and Tables S1 and S2. Data show the mean±s.e.m. Two-tailed unpaired Student's *t*-test was used. *P<0.05, **P<0.01, ***P<0.001.

Given the prevalence of sphingolipid storage across sphingolipidoses (Özkara, 2004) and the significance of storage material accumulation as a major contributor toward organ damage (Platt et al., 2018), we used unbiased lipidomics to examine potential metabolic alterations in the absence of Psap. Lipids from brains isolated from WT siblings and *psap^+63/+63^* or *psap^−14/−14^* zebrafish at the end stage of disease (∼4 mpf), characterized by significantly impaired locomotion and/or cachexia, were extracted using the Bligh–Dyer method (Bligh and Dyer, 1959) for liquid chromatography–mass spectrometry (LC–MS) analysis. In agreement with clinical presentations of lipid storage (Platt et al., 2018), LC–MS identified marked elevations across several sphingolipid classes (Fig. 2A) in both *psap* KO lines (Fig. 2B; Fig. S4A, Tables S1 and S2). The largest increase in lipid content was found in lactosylceramide and hexosylsphingosine (Fig. 2B; Fig. S4A, Tables S1 and S2); chronic accumulations of glucosyl and galactosylsphingosine (psychosine) are associated with the pathogenesis of Gaucher (Lelieveld et al., 2019, 2022) and Krabbe disease (Hawkins-Salsbury et al., 2013; Folts et al., 2016), respectively (the current study reports total hexosylsphingosine, which is likely to be a combination of both glucosyl and galactosyl species). Additional storage material accumulation was consistently observed for ceramide and sphingomyelin (Fig. 2B; Fig. S4A, Tables S1 and S2).

Interestingly, lipid storage in the case of hexosylceramide, ceramide and sphingomyelin was primarily restricted to shorter acyl chain species, with a rapid drop in the degree of lipid accumulation occurring at around 40 carbon atoms (Fig. 2C; Fig. S4B), corresponding to a C22 acyl chain for the majority of sphingolipids given prevalence of the d18 backbone in adult zebrafish brains (Zhang et al., 2019). A similar trend of acyl chain-dependent ceramide alterations was previously identified in a zebrafish model of Farber disease (Zhang et al., 2019). Taken together, these findings suggest that saposins, and potentially additional enzymes involved in sphingolipidoses, may regulate the catabolism of different sphingolipid subspecies differently. As fatty acid degradation is length and organelle specific [short- and medium-chain fatty acids undergo mitochondrial breakdown, whereas very long chain (∼C22 and higher) and branched-chain species undergo peroxisomal breakdown (Stradomska et al., 2020)], the sharp drop in lipid accumulation at C22 in the *psap* KO model could also be an indicator of organelle-specific dysfunction. Continued exploration of organelle-specific metabolism in the absence of *psap* could shed further light on this question.

### *psap* KO zebrafish exhibit impaired locomotion, storage material accumulation and myelin loss without significant loss of oligodendrocyte markers

**Fig. 3.**
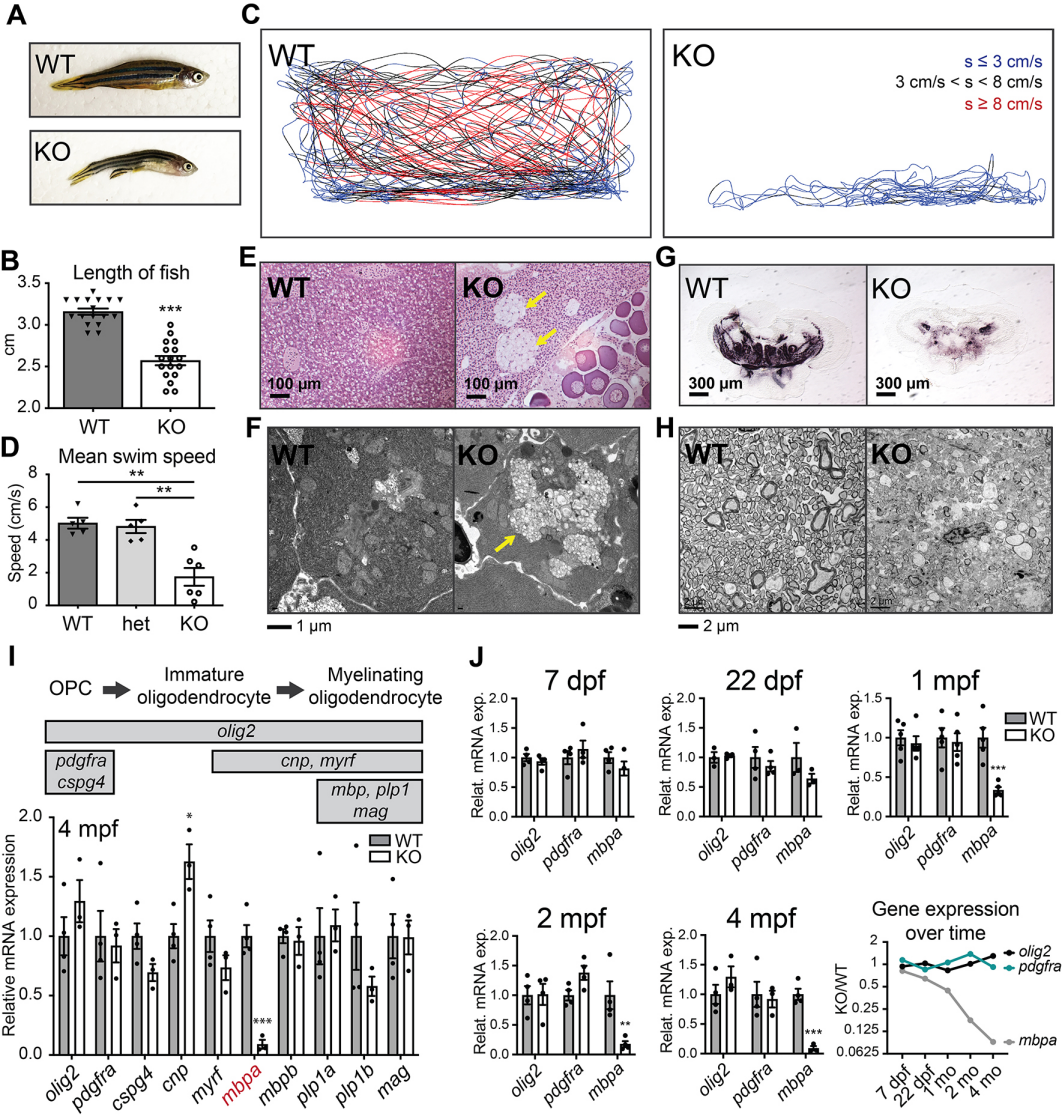
***Psap* KO zebrafish exhibit impaired locomotion, storage material accumulation and myelin loss without significant loss of oligodendrocyte markers.** (A) Representative images of a 4 mpf *psap^+63/+63^* zebrafish and WT sibling. Length distributions of 4 mpf WT and *psap* KO zebrafish are shown in B. (B) Length measurements of 4 mpf *psap^+63/+63^* zebrafish and WT siblings. *n*=17 WT, 17 *psap^+63/+63^* fish. A decreased length was observed in nearly all *psap^+63/+63^* and *psap^−14/−14^* zebrafish for each generation examined. (C) Swim behavior tracking for 4 mpf *psap^−14/−14^* and WT zebrafish. Disrupted locomotion was present in all *psap^+63/+63^* and *psap^−14/−14^* zebrafish for each generation examined. Representative data from five WT and six *psap^−14/−14^* zebrafish. s, swim speed. Zebrafish were filmed from the side of a 2.75-l fish tank. The filming area encompasses the entire side view of the tank and is ∼28 cm in length and 13 cm in height. (D) Mean swim speed of 4 mpf WT, *psap^−14/+^* and *psap^−14/−14^* zebrafish. (E) H&E staining of 4 mpf *psap^+63/+63^* and WT zebrafish liver sections. Representative data from three WT, one *psap^−14/−14^* and three *psap^+63/+63^* zebrafish. Arrows point to regions containing large, foamy cell clusters. (F) Transmission electron microscopy (TEM) of 4 mpf *psap^+63/+63^* and WT zebrafish livers. The arrow indicates regions of storage material accumulation. Representative data from three WT and three *psap^+63/+63^* zebrafish. (G) Black Gold II staining of frozen brain sections from 4 mpf *psap^+63/+63^* zebrafish and WT siblings. Representative data from five WT and five *psap^+63/+63^* zebrafish. Additional serial sections are in Fig. S5, and several brain sections from G are also reproduced among the sequential sections in Fig. S5. (H) TEM of optic nerve sections from 4 mpf *psap^+63/+63^* zebrafish and WT siblings. Representative data from five WT and six *psap^+63/+63^* zebrafish. (I) Schematic of the expression of stage-specific marker genes (top). qRT-PCR of major oligodendrocyte differentiation markers in 4 mpf *psap^+63/+63^* and WT sibling zebrafish brains (bottom). Representative data from two independent datasets; *n*=4 WT and 4 *psap^+63/+63^* for each dataset. (J) qRT-PCR of *olig2*, *pdgfra* and *mbpa* over the course of *psap^+63/+63^* and WT sibling zebrafish brain development. Representative data from at least two independent datasets for each time point; *n*=3-5 WT and 3-5 *psap^+63/+63^* for each dataset. Data show the mean±s.e.m. For B,D,I,J, two-tailed unpaired Student's *t*-test was used. *P<0.05; **P<0.01, ***P<0.001.

Extensive sphingolipid accumulation in the *psap* KO brain raises the question of whether these metabolic changes will translate to pathologies at the tissue and organism level. To address this question, phenotypic characterization was undertaken for both *psap* KO lines. Relative to WT siblings, *psap* KO zebrafish exhibited a reduced size that became apparent after 3 mpf (Fig. 3A,B). Swimming, shoaling and feeding behaviors appeared unaltered within the first ∼3.75 months of life. Between 4 and 5 mpf, all *psap* KO zebrafish exhibited a rapid decline in locomotor ability, characterized by increasing difficulty maintaining neutral buoyancy and decreased swim speed (Fig. 3C,D); euthanasia was typically necessary by 4.5 mpf, with no survival past 5 mpf. In agreement with the autosomal recessive pattern of inheritance for nearly all sphingolipidoses (Platt et al., 2018), no behavioral change or early mortality was observed in the heterozygous population (Fig. 3D). Given the similarity in the time of onset and disease severity between the *psap^+63/+63^* and *psap^−14/−14^* lines, all subsequent studies were performed with *psap^+63/+63^* zebrafish and WT siblings.

To evaluate potential changes in tissue morphology, 4 mpf *psap^+63/+63^* and WT zebrafish were fixed, embedded in JB-4 resin, sectioned, and stained with Hematoxylin and Eosin (H&E). Histological evaluation of *psap^+63/+63^* and WT fish revealed the presence of large, foamy cell clusters in the livers of the former (Fig. 3E), which has also been reported in zebrafish models of Gaucher disease (Keatinge et al., 2015; Lelieveld et al., 2019). Storage material was also clearly visible within individual hepatocytes by transmission electron microscopy (TEM) (Fig. 3F). CNS myelination in 4 mpf *psap^+63/+63^* and WT zebrafish was examined using two separate approaches: histochemical staining of myelin in frozen brain sections and TEM of optic nerves. Serial frozen *psap^+63/+63^* brain sections stained for myelin with Black Gold II (Schmued et al., 2008) showed a marked reduction in myelin levels (Fig. 3G), with myelin loss prevalent across most brain regions (Fig. S5). Significant myelin loss was also observed in *psap^+63/+63^* optic nerves via TEM (Fig. 3H).

Within the vertebrate CNS, oligodendrocytes (OLs) are the myelinating glial population indispensable for neuron health (Emery, 2010; Kuhn et al., 2019). During neural development, cells of the OL lineage differentiate from OL precursor cells (OPCs) to premyelinating and then mature myelinating OLs, characterized by defined changes in morphology and expression of stage-specific marker genes (Fig. 3I, top panel) (Emery, 2010; Kuhn et al., 2019). Given the severe myelin loss observed in our model, we hypothesized that the OL differentiation program might be adversely affected by the absence of Psap. To test this hypothesis, quantitative real-time PCR (qRT-PCR) was performed on 4 mpf WT and *psap^+63/+63^* brains, focusing on the expression of the OPC markers *pdgfra* and *cspg4*, the OL differentiation markers *cnp* and *myrf*, and the myelinating OL markers *mbpa*, *mbpb*, *plp1a*, *plp1b* and *mag* (Fig. 3I, top panel) (Pepper et al., 2018). The OL lineage marker *olig2*, which is expressed across all stages of OL development and has been shown to be a reliable indicator of absolute OL number in the mouse brain (Valério-Gomes et al., 2018), was also included in the analysis. Surprisingly, with the exception of *mbpa* (*myelin basic protein a*), the expression of which was significantly reduced in *psap^+63/+63^* brains, expression of the other markers of OL development was largely unperturbed even at the end stage of disease (Fig. 3I). Loss of *mbpa* expression first appeared at ∼1 mpf and progressively decreased over time, whereas expression of the OPC and OL lineage markers *pdgfra* and *olig2* remained unchanged over the entire course of disease progression (Fig. 3J), implicating OL dysfunction, rather than massive OL loss, as a more likely contributor toward demyelination in the *psap* KO model.

### Brain transcriptomics identifies upregulated inflammation and mTORC1 signaling in *psap^+63/+63^* zebrafish

**Fig. 4.**
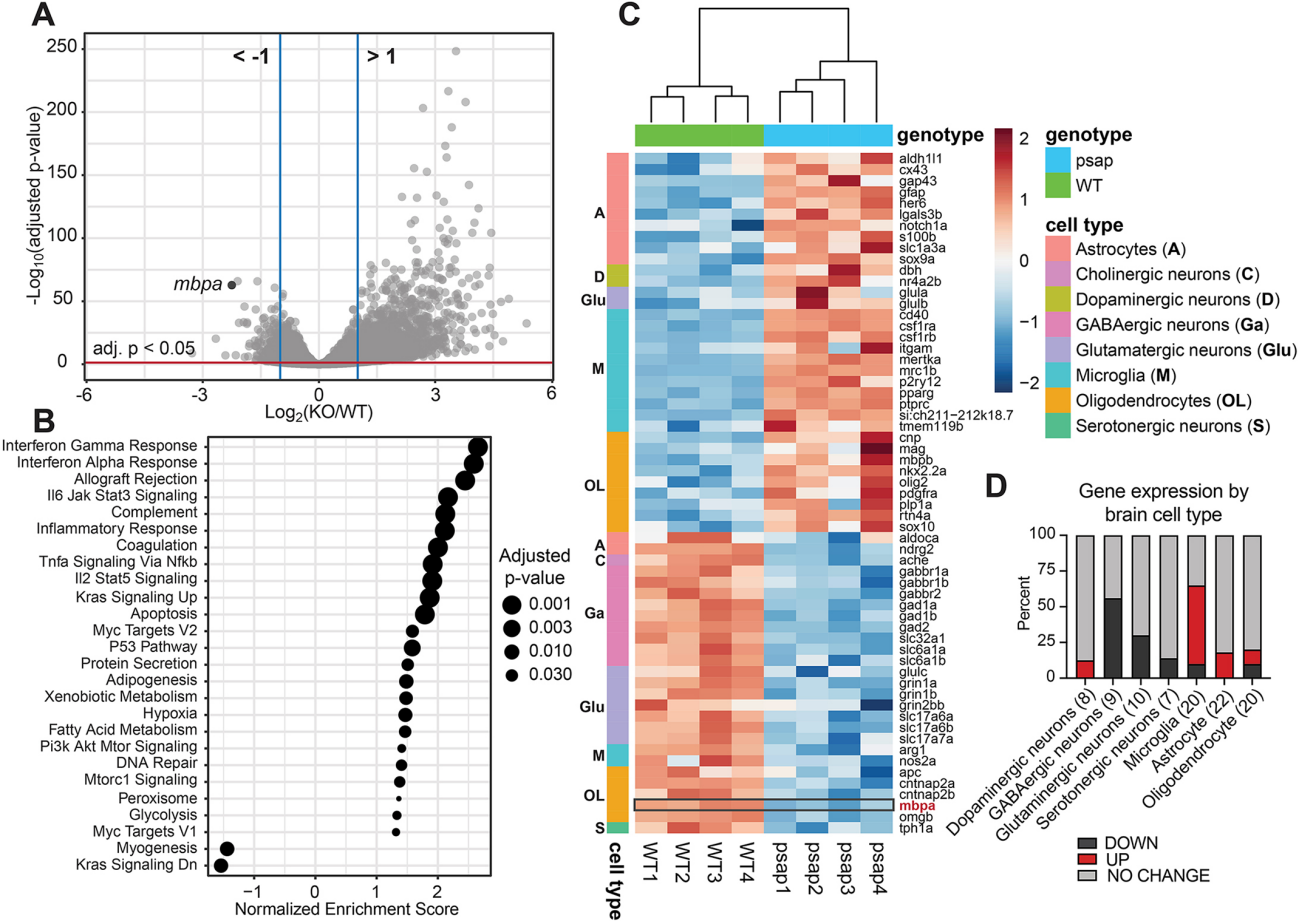
**Brain transcriptomics identifies upregulated inflammation and mTORC1 signaling in *psap^+63/+63^* zebrafish.** (A) Volcano plot of all annotated genes from transcriptomics analysis of 4 mpf WT (*n*=4) and *psap^+63/+63^* (*n*=4) zebrafish brains. *mbpa* is indicated on graph. Differential expression analysis data are in Table S3. (B) Gene set enrichment analysis (GSEA) of transcriptomics data (all genes from Table S3) using the Hallmark gene sets (MSigDB Collections). Additional GSEA analyses are in Table S4. (C) Hierarchical clustering analysis of WT and *psap^+63/+63^* RNA sequencing data based on a manually curated list of 98 brain cell-type-specific marker genes (Table S5). The graph displays the 61 significantly deregulated genes (adjusted *P*-value <0.05) from the curated list. The boxed region corresponds to *mbpa* (third gene from bottom). (D) Percentage of up- and down-regulated brain cell type-specific marker genes (Table S5). Numbers in parentheses denote the number of genes in each cell type. ‘UP’ and ‘DOWN’ denote statistically significant (adjusted *P*-value <0.05) genes for which the relative (KO/WT) expression >1.5 and <0.67, respectively; all additional genes are designated ‘NO CHANGE’.

To uncover pathways that drive *mbpa* and myelin loss in the *psap* KO model, brain transcriptomics was performed on *psap^+63/+63^* zebrafish and WT siblings. Brains from adult zebrafish at 4 mpf were processed for RNA sequencing, pooling three brains per sample to ensure sufficient materials for analysis. Data analysis using DESeq2 (Love et al., 2014) identified 22,871 genes, 4659 (20.4%) of which were upregulated in *psap* KO brains, whereas 4359 (19.1%) were downregulated (Fig. 4A; Table S3). A dot plot of all significantly enriched pathways from gene set enrichment analysis (GSEA) using the Molecular Signatures Database Hallmark Gene Set Collection (Liberzon et al., 2015) is shown in Fig. 4B (additional pathway analysis data are provided in Table S4). In agreement with the prevalence of neuroinflammation across many LSDs (Platt et al., 2018), the highly significantly upregulated pathways are dominated by those involved in the inflammatory response (Fig. 4B; Table S4). mTORC1 signaling, a major modulator of myelination (Figlia et al., 2018), was also among the upregulated pathways in the Hallmark GSEA (Fig. 4B). Notably, differential gene expression analysis (Table S3, ‘psap_vs_WT’) also revealed *gpnmb* (*glycoprotein nonmetastatic melanoma protein b*) to be one of the most upregulated (∼30-fold) genes in *psap* KO brains. *gpnmb* expression is elevated in the livers of Gaucher disease zebrafish (Lelieveld et al., 2022), and gpNMB has been identified as a marker for glucosylceramide-laden macrophages in both Gaucher disease patients and mice (Kramer et al., 2016).

To gauge how distinct brain cell populations may be affected in the context of the altered pathways, hierarchical clustering was performed using a manually curated list of gene markers representing astrocytes, microglia, OLs and different types of neurons (Table S5). Mirroring the overrepresentation of inflammatory pathways in the GSEA, clustering analysis revealed preferential sorting of microglia and astrocyte markers into the *psap* KO-upregulated cluster, and neuron markers into the *psap* KO-downregulated cluster (Fig. 4C,D), indicating microglia/astrocyte activation and neuron loss. In agreement with prior qRT-PCR results, OL marker expression levels were not highly altered between WT and *psap^+63/+63^* brains (Fig. 4D), suggesting that cells of the OL lineage are not appreciably different in number or differentiation following loss of Psap, despite reduced *mbpa* expression. Meanwhile, overrepresentation of the ‘apoptosis’ gene set (Fig. 4B) could be attributed to neuronal apoptosis.

### Disrupted mTORC1 signaling does not precede *mbpa* loss in *psap^+63/+63^* zebrafish

Although the upregulation of mTORC1 and proinflammatory signaling in the *psap* KO brain supports the targeting of these pathways for sphingolipidosis treatment, the current RNA sequencing was performed at the symptomatic end stage of disease and did not yield temporal information on the relative occurrences of disease-associated events. For anti-inflammatory and/or mTORC1 modulatory compounds to be efficacious in the treatment of neurodegeneration, it is crucial that the onset of inflammation and mTORC1 dysregulation precede or coincide with *mbpa* loss. Examination of these cellular events over the entire course of disease progression could yield valuable information on the feasibility of applying the aforementioned compounds to the *psap* KO model, while also defining the optimal timeframe for treatment.

**Fig. 5.**
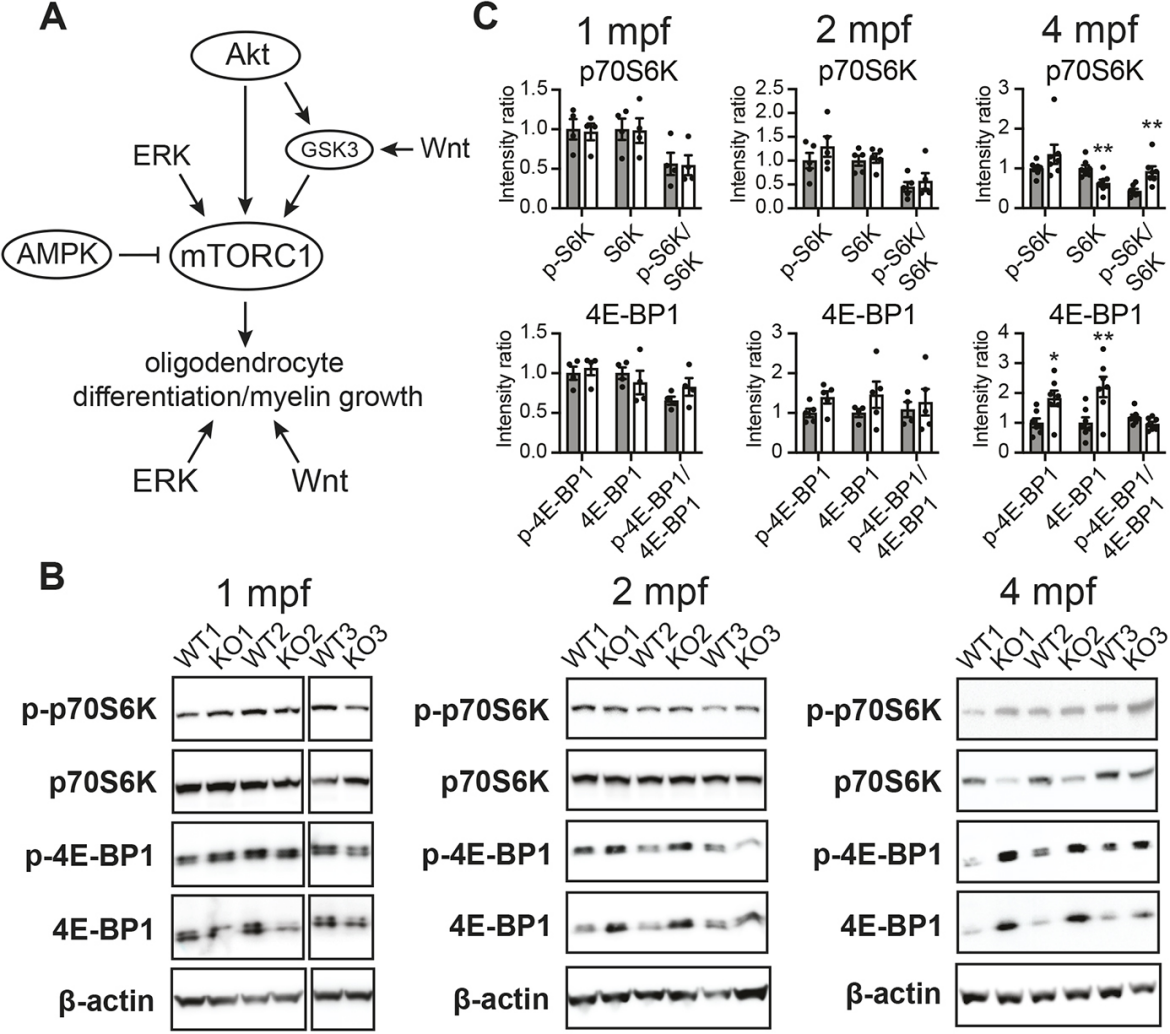
**Disrupted mTORC1 signaling does not precede *mbpa* loss in *psap^+63/+63^* zebrafish.** (A) Illustration of the major promyelinating signaling pathways examined in the current study. (B) Western blots of phosphorylated and unphosphorylated p70S6K and 4E-BP1 in *psap^+63/+63^* and WT sibling brains, demonstrating disrupted mTORC1 signaling at 4 mpf but no additional changes. Representative data from *n*=5-14 WT and *n*=4-14 *psap^+63/+63^* zebrafish; the signaling status was monitored over three independently propagated generations of fish to yield comparable results. Data for additional pathways illustrated in A are in Fig. S6. (C) Densitometry calculations of p-p70S6K, p70S6K, p-4E-BP1, 4E-BP1, and phosphorylated/non-phosphorylated protein ratios for 1 mpf, 2 mpf and 4 mpf *psap^+63/+63^* zebrafish and WT siblings. Data points for each dataset were normalized to the average of the WT samples in that set. Data show the mean±s.e.m. Two-tailed unpaired Student's *t*-test was used. *P<0.05, **P<0.01.

OL differentiation, myelination and remyelination are controlled by a series of complex and well-conserved intracellular signaling pathways (Fig. 5A) (Gaesser and Fyffe-Maricich, 2016). To determine whether disruptions in these pathways contribute to *mbpa* loss in the *psap* KO brain, we used western blotting to probe the activation status of the major myelin modulatory pathways Akt/mTOR, ERK1/2, AMPK and Wnt (additional hypoxia-responsive pathways were not examined owing to the absence of zebrafish-specific antibodies) (Gaesser and Fyffe-Maricich, 2016). With the exception of mTORC1 substrate perturbations at the end stage of disease, the expression levels of proteins involved in most of these signaling pathways were unaltered between WT and *psap^+63/+63^* brains (Fig. S6). Both the non-phosphorylated (p-) and phosphorylated forms of the mTORC1 substrate 4E-BP1 (encoded by *eif4ebp1*) were consistently elevated in *psap* KO brains at 4 mpf, but not at 1 and 2 mpf; the p-4E-BP1/4E-BP1 ratio was unchanged for all three timepoints (Fig. 5B,C). In the case of the mTORC1 substrate p70S6K (encoded by *rps6kb1*), an increased p-p70S6K/p70S6K ratio was observed at 4 mpf owing to decreased levels of unphosphorylated p70S6K, but no changes in p-p70S6K or p70S6K were detected at 1 and 2 mpf (Fig. 5B,C). Although altered levels of 4E-BP1 and p70S6K are likely indicators of dysregulated mTORC1 signaling, the absence of changes in protein levels at the onset of *mbpa* loss (1 mpf) suggests that aberrant mTORC1 signaling is unlikely to be a direct cause of demyelination. Instead, the lysosomal localization of mTORC1 components (Rabanal-Ruiz and Korolchuk, 2018) suggests that disrupted mTORC1 signaling may be the consequence of altered lysosomal morphology at a late stage of disease.

### Neuroinflammatory response precedes *mbpa* loss in *psap^+63/+63^* zebrafish

**Fig. 6.**
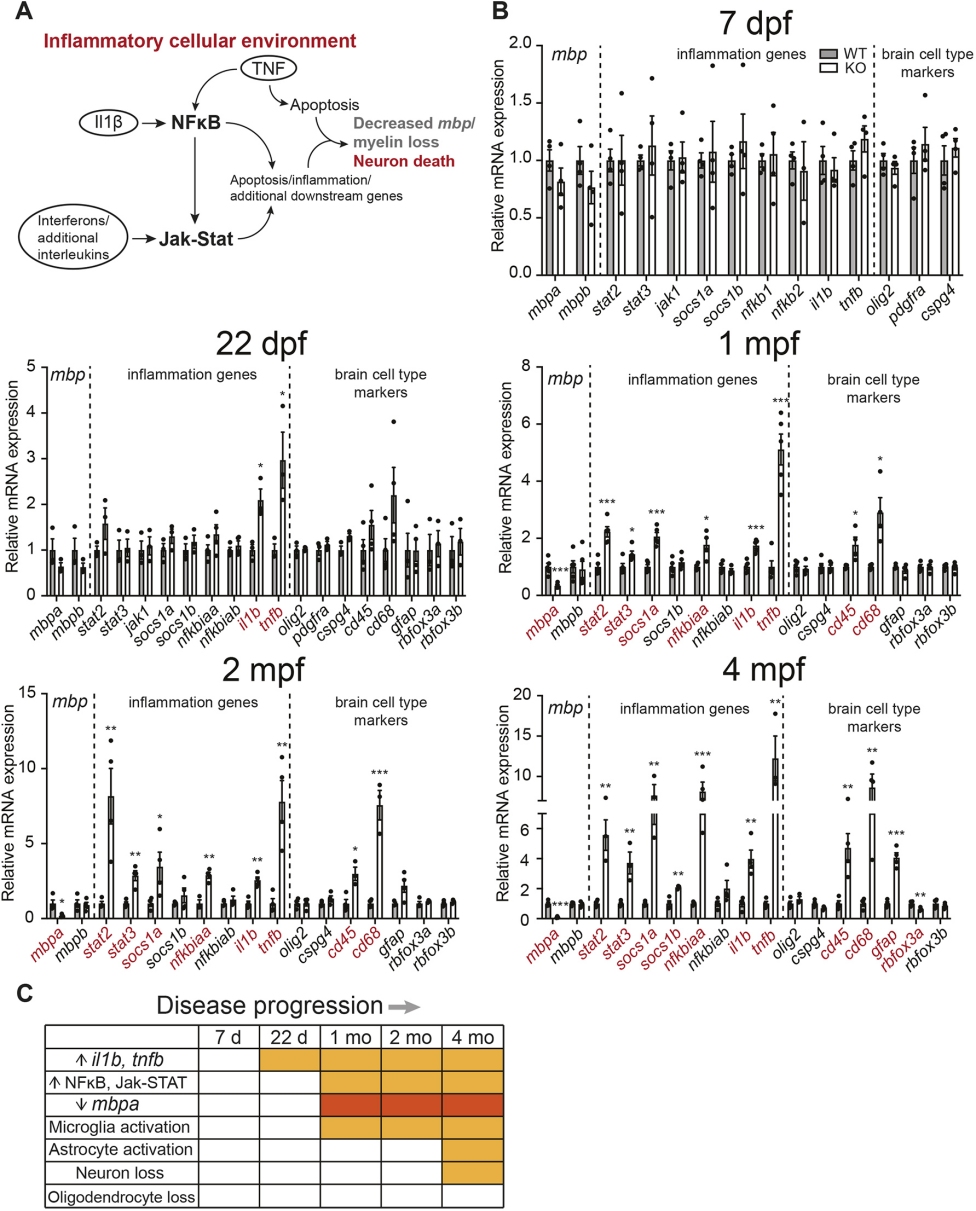
**Neuroinflammatory response precedes *mbpa* loss in *psap^+63/+63^* zebrafish.** (A) Schematic of inflammation-driven disease progression in the *psap* KO model. (B) Relative expressions of representative markers for myelination, inflammation and major brain cell populations at 7 dpf, 22 dpf, 1 mpf (33 dpf), 2 mpf and 4 mpf. Representative data from two independent qRT-PCR datasets; *n*=3-5 WT and *n*=3-5 *psap^+63/+63^* for each dataset. Data points for each dataset were normalized to the average of the WT samples in that set. Data show the mean±s.e.m. Two-tailed unpaired Student's *t*-test was used. *P<0.05; **P<0.01, ***P<0.001. (C) Table illustrating the onset of major cellular events in *psap^+63/+63^* zebrafish relative to WT siblings, based on first appearance of statistically significant changes in the corresponding marker genes. Red boxes denote statistically significant time points for *mbpa*; orange boxes denote statistically significant time points for all additional pathway markers.

A closer examination of the pathways from Fig. 4B via the Reactome Pathway Database (Gillespie et al., 2022) suggests that heightened inflammatory response in the absence of Psap is likely to funnel through NFκB and Jak-Stat signaling (Fig. 6A); persistent upregulation in these pathways creates a highly inflammatory cellular environment that may in turn disrupt OL myelination and neuronal health. To determine whether an elevated neuroinflammatory response preceded *mbpa* loss in the *psap* KO model, 7 days-post-fertilization (dpf) larvae and brains from 22 dpf, 1 mpf (33 dpf), 2 mpf and 4 mpf WT and *psap^+63/+63^* zebrafish were isolated and processed for qRT-PCR, focusing on the following genes: *mbpa* and *mbpb* (myelination); *nfkb1*, *nfkb2*, *nfkbiaa* and *nfkbiab* (NFκB signaling); *stat2*, *stat3*, *jak1*, *socs1a* and *socs1b* (Jak-Stat signaling); *tnfb* and *il1b* (proinflammatory cytokines); *olig2* (OL lineage); *pdfgra* and *cspg4* (OPCs); *cd45* (also known as *ptprc*) and *cd68* (microglia); *gfap* (astrocytes); and *rbfox3a* and *rbfox3b* (neurons). qRT-PCR was used to examine NFκB and Jak-Stat signaling owing to low epitope conservation between major mammalian and zebrafish NFκB pathway components, which hindered antibody-based detection (O'Leary et al., 2016). Although the lack of protein-based detection may delay visualization of celllular events associated with rapid phosphorylation, the inclusion of multiple negative regulators (*socs1a*, *socs1b*, *nfkbiaa* and *nfkbiab*) that are sensitive to NFκB/Jak-Stat overactivation in the qRT-PCR analysis will help improve the accuracy of pathway dynamics prediction.

Detailed qRT-PCR data for the aforementioned genes are in Fig. 6B (statistically significant gene labels in red), and a summary of the onset of associated cellular events is in Fig. 6C. Within the *psap* KO brain, rise in proinflammatory cytokines (22 dpf) preceded NFκB, Jak-Stat and microglia activation, which coincided with *mbpa* loss at ∼1 mpf. Notably, astrocyte activation and neuron loss, as demonstrated by changes in *gfap*, *rbfox3a* and *rbfox3b* expression, did not occur until the later stages of disease (Fig. 6B,C), coinciding with the appearance of locomotion impairment at ∼4 mpf (Fig. 3C). The expression of the major markers of OPCs and the OL lineage remained consistent over the entire course of disease progression (Fig. 6B,C). Taken together, these data indicate that an inflammatory cellular environment in the absence of Psap precedes failed OL myelination and neuron loss, and supports the inhibition of proinflammatory signaling as a potential route toward improving CNS health in the *psap* KO model.

### Two strategies toward therapy in the *psap* KO model

Having established and characterized a zebrafish model of combined saposin deficiency, we hoped to use our results as the basis for treatment testing. The early rise in NFκB and Jak-Stat signaling in the *psap* KO brain supports pharmacological modulation of these pathways as one strategy toward symptom alleviation. Additionally, given recent evidence of successful Farber and Gaucher disease rescue via acid sphingomyelinase knockout (Keatinge et al., 2023; Beckmann et al., 2019), we also examined this genetic rescue strategy for the *psap* KO model.

**Fig. 7.**
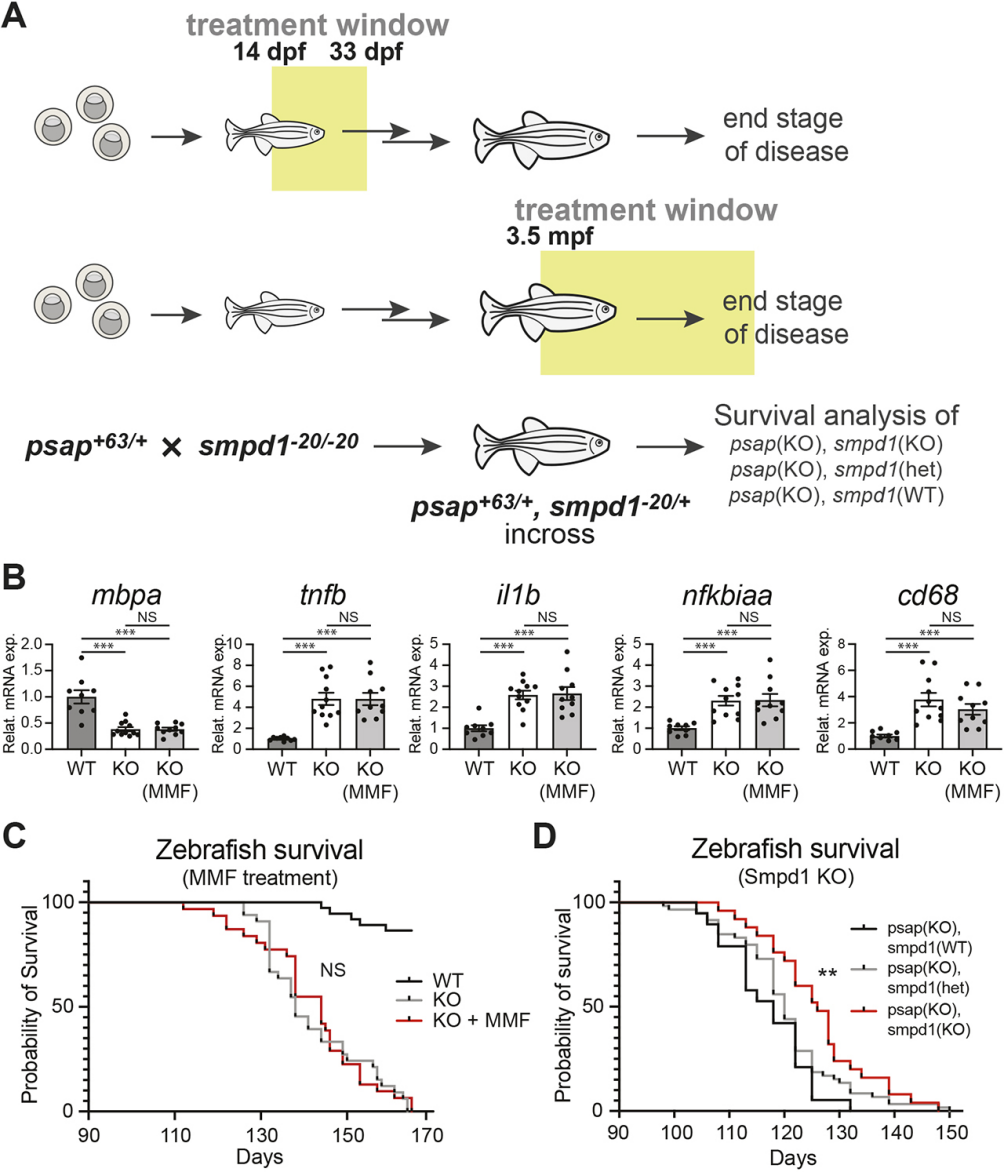
**Two strategies toward therapy in the *psap* KO model.** (A) Three treatment schemes in the *psap* KO model. (B) qRT-PCR of *mbpa*, *tnfb*, *il1b*, *nfkbiaa* and *cd68* in 33 dpf WT (*n*=9), untreated *psap^+63/+63^* (*n*=11), and MMF-treated *psap^+63/+63^* (*n*=10) zebrafish brains. Data show the mean±s.e.m. Two-tailed unpaired Student's *t*-test was used. NS, not significant; ***P<0.001. (C) Survival analysis of WT (*n*=37), untreated *psap^+63/+63^* (*n*=33) and MMF-treated *psap^+63/+63^* (*n*=31) zebrafish starting from 3 mpf. Log-rank (Mantel–Cox) test was used. Results were not significantly different for KO versus KO+MMF; *P*<0.001 for WT versus KO and WT versus KO+MMF. (D) Survival analysis of *psap^+63/+63^, smpd1^+/+^* (KO/WT, black line) (*n*=19); *psap^+63/+63^, smpd1^−20/+^* (KO/het, gray line) (*n*=59); and *psap^+63/+63^*, *smpd1^−20/−20^* (KO/KO, red line) (*n*=25) zebrafish starting from 3 mpf. Log-rank (Mantel–Cox) test was used. Results were not significantly different for KO/WT versus KO/het and KO/KO versus KO/het. **P<0.01 for KO/WT vs KO/KO.

A survey of the existing literature uncovered over 50 candidate compounds for NFκB/Jak-Stat inhibition, which were filtered based on the criteria of (1) blood-brain barrier permeability, (2) aqueous solubility and (3) established safety profile and/or US Food and Drug Administration (FDA) approval to obtain the two testing compounds tofacitinib (Xeljanz) (Dhillon, 2017) and monomethylfumarate (MMF; Bafiertam) (Berger et al., 2021). Tofacitinib is a nanomolar inhibitor of Jak1-3 (Changelian et al., 2003) and MMF is a modulator of multiple cellular pathways including NFκB (Parodi et al., 2015; Mazzola et al., 2017) and Nrf2 (Linker et al., 2011); these compounds are FDA-approved for the treatment of rheumatoid arthritis and multiple sclerosis, respectively (Dhillon, 2017; Berger et al., 2021). Although cotreatment was initially considered, recent reports of demyelination in two patients undergoing tofacitinib therapy suggest that simultaneous inhibition of Jak1-3 may unintentionally disrupt myelin-associated pathways (Eckert et al., 2019; Massoud et al., 2020). Consequently, MMF was examined as a potential monotherapy in the *psap* KO model. Following initial testing, during which reduced locomotion was observed in a subpopulation of MMF-treated *psap^+63/+63^* adult zebrafish at 200 µM (data not shown), 100 µM was established as the dosage for long-term treatment. Based on the temporal expression patterns of myelination- and inflammation-specific genes (Fig. 6B,C), we designed two separate treatment schemes in which *psap^+63/+63^* zebrafish are treated either at the juvenile stage from 14 to 33 dpf, or at the adult stage from 3.5 mpf until the time of euthanasia owing to the severity of the disease (Fig. 7A). MMF treatment of juvenile and adult zebrafish did not alter gene expression (Fig. 7B) or prolong lifespan (Fig. 7C).

**Fig. 8.**
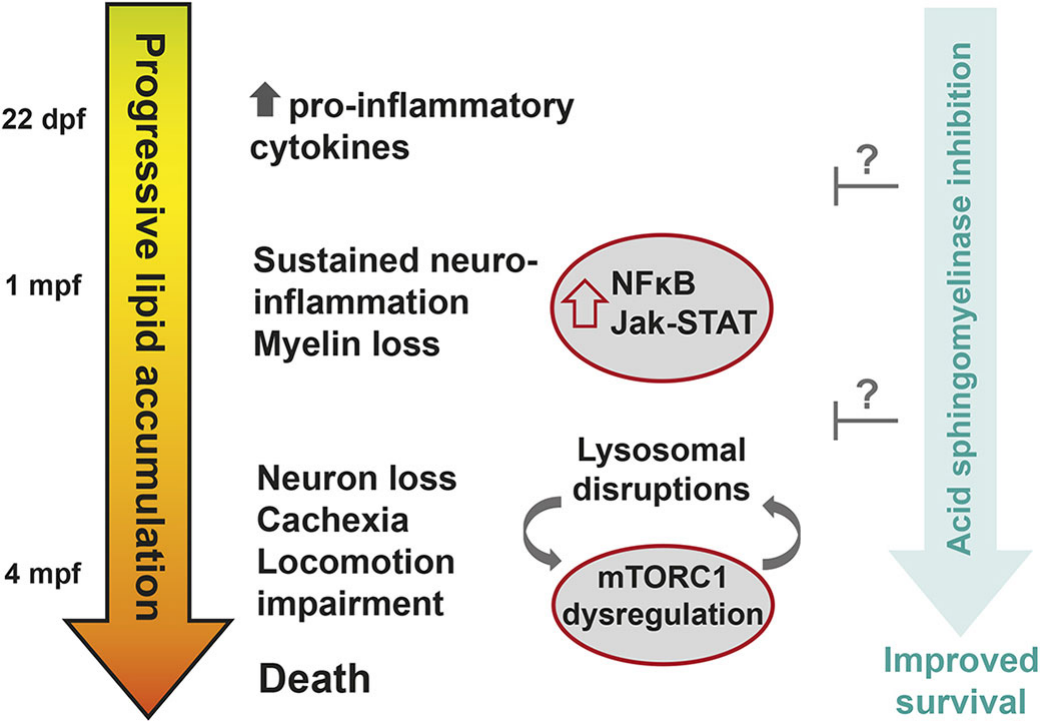
**Schematic of the proposed sequence of events driving disease progression in the combined saposin deficiency zebrafish model.** Increased *tnfb* and *il1b* expression is detected at ∼22 dpf in the *psap^−/−^* brain, followed by increased expression of additional neuroinflammatory markers (*stat2*, *stat3*, *socs1a*, *nfkbiaa*, *cd45* and *cd68*) and loss of myelin (reduced *mbpa*) at ∼1 mpf; these pathologies worsen over time, preceding loss of neuronal markers (*gfap*, *rbfox3a*) and dysregulated mTORC1 signaling at ∼4 mpf, fairly rapid onset of cachexia and locomotion impairment within 2 weeks around the 4-mpf time point, and death of all *psap^−/−^* fish by 5 mpf. Knockout of acid sphingomyelinase (*smpd1*) in *psap^−/−^* zebrafish leads to a modest increase in lifespan via yet to be investigated mechanisms.

In addition to small-molecule-based treatment, a genetic rescue was also explored based on previous reports of successful Farber and Gaucher disease mitigation by crossing the respective mouse and zebrafish models with an acid sphingomyelinase (SMPD1) deficiency model (Keatinge et al., 2020 preprint; Beckmann et al., 2019). In the context of the sphingolipid metabolic pathway, it is possible that SMPD1 knockout-induced ceramide depletion may counter ceramide accumulation in Farber disease and enhance lifespan in the Gaucher model by shifting complex sphingolipid catabolism toward ceramide instead of psychosine. To examine the effect of *smpd1* mutagenesis on the *psap* KO model, we generated doubly heterozygous (*psap^+63/+^, smpd1^−20/+^*) zebrafish (Fig. 7A; Fig. S7), which were incrossed and monitored for survival overtime. Although all fish with reduced lifespan were homozygous at the *psap* allele, doubly homozygous zebrafish exhibited a modest increase in survival relative to that of *psap^+63/+63^, smpd1^−20/+^* and *psap^+63/+63^, smpd1^+/+^* siblings (Fig. 7D). Taken together, the availability of zebrafish models of combined saposin deficiency and additional LSDs has enabled the examination of pathways involved in disease progression, as well as pharmacological and genetic approaches toward therapy (Fig. 8). Our findings highlight the modulation of acid sphingomyelinase activity as a potential path toward the treatment of multiple sphingolipidoses.

## DISCUSSION

The current study presents the first known model of combined saposin deficiency in the zebrafish. CRISPR-Cas9 targeting of an early exon preceding all major saposin domains resulted in a nonsense *psap* mutant that recapitulated both the CNS and peripheral pathologies associated with saposin loss. Notably, *psap* mutagenesis did not lead to nonsense-mediated mRNA decay based on transcriptomics analysis. Given the ability of most sphingolipids to undergo interconversions via the sphingolipid metabolic pathway (Qin et al., 2010), *psap* mutagenesis triggered the upregulation of additional lysosomal sphingolipid catabolic genes, which failed to rescue lipid storage in this model, likely owing to the synergy between saposins and sphingolipid catabolic enzymes that is necessary for sphingolipid breakdown (Özkara, 2004). Importantly, loss of individual saposins in humans leads to saposin-specific clinical presentations (Kuchar et al., 2009; Kang et al., 2018; Hulková et al., 2001), as SapA-SapD exhibit distinct preferences for specific sphingolipid catabolic enzymes (Özkara, 2004). Although the combined saposin deficiency model was intended to capture the most severe form of saposin loss, studies with single saposin-knockout models could yield additional insight on saposin-type-dependent disease mechanisms.

Given the prevalence and severity of myelin loss across many LSDs (Folkerth, 1999), we chose to closely characterize this aspect of neurodevelopment in the *psap* KO model. Histochemical staining and TEM revealed severe demyelination within the optic nerves and across the entire *psap* KO brain. Myelin loss was driven at least in part by changes on the transcriptional level, as *psap* KO zebrafish exhibited progressive loss of the *mbpa* mRNA starting from ∼1 mpf. Surprisingly, expression of the OL lineage marker *olig2* and OPC markers *pdgfra* and *cspg4* remained unaffected over the entire course (7 dpf-4 mpf) of disease progression. The expression of additional markers of OL maturation, including *cnp*, *myrf*, *plp1a*, *plp1b* and *mag*, was also unchanged at the end stage of disease, whereas multiple neuronal markers were downregulated, suggesting that the OL lineage is unlikely to be the predominant brain cell type undergoing cell death in the *psap* KO model.

OL loss has been reported across multiple LSD models (Fletcher et al., 2014, 2011; Prolo et al., 2009; Parente et al., 2012). A detailed examination of OL status in a canine model of fucosidosis identified significant OL loss in the corpus callosum and cerebellar white matter, which stabilized by 16 weeks of age, as visualized by decreased *CNP*, *MAG*, *MAL* and *PLP1* expression, and reduced CNP and increased CASP6 staining (Fletcher et al., 2014). Decreased numbers of optic nerve mature OLs, but not OPCs, were observed in a mouse model of sialin deficiency (Prolo et al., 2009), whereas brain region-specific transcriptomics in a mouse mucopolysaccharidosis VII model revealed changes in the expression of 53% of examined OL markers (Cahoy et al., 2008), all of which were downregulated (Parente et al., 2012). Notably, the expression of the majority of the previously reported OL markers was unaltered in our zebrafish model, highlighting the variability in glial response across different LSDs and host organisms. Importantly, premyelinating OLs with extended processes and dystrophic axons have been identified within chronic lesions from multiple sclerosis patients, suggesting that hindered OL-axon interaction, rather than the absence of OLs, was the major limiting factor toward remyelination in multiple sclerosis (Chang et al., 2002). Although our qRT-PCR data do not provide morphological confirmation of OL status within the *psap* KO brain, consistent expression of myelin regulatory factor (*myrf*) and most mature OL markers also supports hindered OL function, rather than OL death, as a more likely driver of myelin loss. Continued evaluation of OLs and neurons via imaging-based approaches will shed light on the detailed morphologies and interactions between these two populations.

The preferential downregulation of *mbpa* in the *psap* KO model also raises the interesting possibility of localized *mbpa* transcript destabilization as a potential mechanism for myelin loss. Unlike most myelin proteins that are translated in the cytoplasm and delivered to the myelin sheath via vesicular transport (Krämer et al., 2001), *mbp* mRNA is transported in RNA granules along microtubules to the site of myelination for local translation, likely to minimize deleterious ectopic protein expression (Krämer et al., 2001; Müller et al., 2013). Among the many molecules involved in Mbp generation, the Src family kinase Fyn is a crucial regulator bridging axon signaling and OL response (Müller et al., 2013). Upon axonal cell adhesion molecule L1 binding to oligodendroglial F3/contactin, activated Fyn phosphorylates multiple downstream targets, including Qki and several heterogeneous nuclear ribonucleoproteins (Hnrnps), leading to *mbp* stabilization and release from RNA granules for translation (Müller et al., 2013; Zhang et al., 2003; White et al., 2008, 2012; Laursen et al., 2011). Given this complex system for Mbp production, disruptions in any modulatory element, possibly owing to delayed OL maturation and/or a highly inflammatory cellular environment, may negatively impact myelin stability. Further investigations of Fyn activity, Hnrnp phosphorylation and microtubule structure within the *psap* KO brain could reveal additional drivers of LSD myelin loss.

To uncover pathways driving demyelination in the *psap* KO model, transcriptomics analysis was conducted on 4 mpf WT and *psap* KO brains to identify upregulation in mTORC1 signaling and multiple inflammatory pathways. mTORC1 is one of the major signaling pathways regulating myelination (Gaesser and Fyffe-Maricich, 2016) and exerts its function over both the active myelin growth and premyelinating stages of development (Figlia et al., 2018). In the CNS, mTORC1 promotes OPC differentiation (Bercury et al., 2014; Zou et al., 2014; Tyler et al., 2009), MBP translation (Bercury et al., 2014; Lebrun-Julien et al., 2014) and *SREBP* transcription (Figlia et al., 2018; Lebrun-Julien et al., 2014), the last leading to increased lipid synthesis for myelin growth. mTORC1 hyperactivation was found to mediate lysosomal dysfunction in an induced pluripotent stem cell model of Gaucher disease (Brown et al., 2019), and rescue of dysregulated mTORC1 signaling in a mouse model of Pompe disease significantly alleviated muscle atrophy (Lim et al., 2017). In the *psap* KO model, dysregulated mTORC1 signaling occurred after *mbpa* reduction, implying that the former, although clearly the result of *psap* mutagenesis, is unlikely to be a direct cause of myelin loss. Importantly, mTORC1 modulates lysosomal biogenesis through phosphorylation of the transcription factor TFEB (Rabanal-Ruiz and Korolchuk, 2018), whereas lysosomal recruitment is required for mTORC1 activation (Roczniak-Ferguson et al., 2012). This crosstalk suggests that dysregulated mTORC1 signaling toward the end stage of saposin deficiency may be due to altered lysosomal morphology; disrupted mTORC1 could in turn perturb lysosomal homeostasis, thus exacerbating pathologies at the lysosome.

Unlike mTORC1 signaling, elevated neuroinflammation preceded *mbpa* loss in the *psap* KO model, such that an increase in the expression of proinflammatory cytokines (∼22 dpf) preceded microglia/NFκB/Jak-Stat activation and *mbpa* loss (∼1 mpf), which preceded astrocyte activation and neuron loss at the late stage of disease (∼4 mpf). The prevalence of neuroinflammation has been well documented across multiple LSD subtypes (Bosch and Kielian, 2015), including sphingolipidoses (Enquist et al., 2007; Potter et al., 2013; Jeyakumar et al., 2003; De Francesco et al., 2013), mucopolysaccharidoses (DiRosario et al., 2009; Villani et al., 2007; Arfi et al., 2011) and neuronal ceroid lipofuscinoses (Macauley et al., 2012; Tarczyluk-Wells et al., 2019; Xiong and Kielian, 2013). A neural progenitor cell-specific mouse model of Gaucher disease exhibited an early increase in the expression of anti-inflammatory cytokines, which shifted to a proinflammatory cytokine profile upon disease progression (Bosch and Kielian, 2015; Enquist et al., 2007; Vitner et al., 2012). Microglia and astrocyte activation was detected in mouse models of Krabbe disease by 2 weeks of age, with microglia activation preceding that of astrocytes and both events preceding demyelination (Potter et al., 2013; Potter and Petryniak, 2016; Sakai et al., 1996; Luzi et al., 2001). Notably, unlike mouse models of Gaucher and Krabbe disease (Tybulewicz et al., 1992; De Gasperi et al., 2004), *psap* KO zebrafish did not exhibit severe phenotypes until ∼4 mpf, with most fish requiring euthanasia by ∼4.5 mpf owing to difficulty maintaining neutral buoyancy and/or cachexia. This delayed but rapid decline raises key questions regarding the cooperation as well as relative impacts of individual cellular events over fatal disease outcomes. As *mbpa* downregulation and microglia activation did not immediately trigger severe symptoms without neuron loss, cell-type-specific disease modeling and detailed imaging of glia-neuron interactions could shed further light on the autonomy of distinct brain cell types in the context of neurodegeneration.

In addition to mechanistic studies, two distinct approaches toward therapy were explored in the *psap* KO model: pharmacological modulation of inflammatory pathways via the FDA-approved multiple sclerosis drug MMF, and genetic rescue via acid sphingomyelinase (*smpd1*) knockout. MMF is the bioactive form of dimethylfumarate (DMF), which is also an approved multiple sclerosis drug with efficacy in reducing MRI lesions and relapse in relapsing-remitting multiple sclerosis patients (Berger et al., 2021; Gold et al., 2020). MMF treatment of juvenile and adult *psap* KO zebrafish did not alter gene expression or prolong lifespan, although it is difficult to rule out the possibility that another treatment regimen might have a different effect. Notably, despite undefined mechanisms of action, DMF and MMF likely act through lipid-derived electrophile (LDE) signaling-like mechanisms, given their structures as derivatives of the electrophilic tricarboxylic acid metabolite fumarate (Poganik and Aye, 2020). Although both DMF and MMF exhibit anti-inflammatory effects via modulation of NFκB and the oxidative stress-responsive transcription factor Nrf2 (Parodi et al., 2015; Mazzola et al., 2017; Linker et al., 2011; Gillard et al., 2015; Kastrati et al., 2016), the large number of potential LDE-modulated targets could widen the range of side effects while diluting impact over LSD-relevant pathways (Poganik and Aye, 2020). Given that gastrointestinal events (nausea, diarrhea and abdominal pain) are a common side effect of DMF (Berger et al., 2021), unanticipated changes in food intake may also exacerbate existing phenotypes in the treatment cohort. Screening of a more extensive list of candidate compounds, along with detailed evaluations of dosage, side effects and alternative delivery methods (Kinkel et al., 2010; Collymore et al., 2013), could help improve the chance of hit compound identification in the *psap* KO model.

Although water tank MMF administration did not alter the phenotype, a modest increase in lifespan was observed upon crossing the *psap* KO line into a zebrafish model of *smpd1* deficiency. This study was inspired by previous reports of increased survival in Farber (*ASAH1*) (Beckmann et al., 2019) and Gaucher (*gba*) (Keatinge et al., 2023) disease animal models following *SMPD1* knockout. In *ASAH1^−/−^* mice, *SMPD1* knockout ameliorated multiple disease pathologies, including ceramide accumulation, peripheral organ histopathology and inflammation (Beckmann et al., 2019). In *gba^−/−^* zebrafish, *smpd1* knockout further raised sphingolipid levels, but rescued mitochondrial respiratory chain function, leading to improved motor behavior and survival (Keatinge et al., 2023). Morphologically, the progressive cachexia and impaired swimming associated with Psap loss more closely resembles the disease course of *gba^−/−^* zebrafish (Keatinge et al., 2015; Lelieveld et al., 2019), rather than the overall reduced size (with no visible cachexia or behavioral change) previously reported in a Farber disease zebrafish model (Zhang et al., 2019). Histologically, the presence of large foamy cells in the *psap^−/−^* liver is reminiscent of the Gaucher cells found in *gba^−/−^* zebrafish, whereas elevated inflammation is present across all three sphingolipidoses (Beckmann et al., 2019; Keatinge et al., 2015; Lelieveld et al., 2019, 2022). Based on these findings, it is possible that Smpd1 inhibition improves the survival of *psap^−/−^* zebrafish by alleviating pathologies shared with the Gaucher and Farber disease models.

Metabolically, as sphingomyelin hydrolysis contributes significantly to the cellular ceramide pool (Sordillo et al., 2016), Smpd1 loss may trigger ceramide depletion and compensation in the form of complex sphingolipid catabolism, thereby hindering the conversion of these species into cytotoxic hexosylsphingosines. Notably, reduced glucosylsphingosine accumulation, improved locomotion and enhanced survival have also been observed in *gba^−/−^* zebrafish following *asah1b* knockout, with Asah1b, but not Asah1a, contributing to glucosylsphingosine production in the absence of Gba (Lelieveld et al., 2022). Given the interconnectedness of sphingolipid catabolic pathways, these findings suggest that the modulation of enzymes surrounding the primary sphingolipidosis-associated enzyme could be an effective approach toward therapy, by shifting sphingolipid metabolism to disfavor the generation of harmful metabolites.

Finally, it is important to note that although SMPD1 loss leads to sphingolipidosis, elevated SMPD1 is also linked to multiple neurological and metabolic disorders (Kornhuber et al., 2010; Mameli et al., 2022). Consequently, a variety of SMPD1 inhibitors, including cationic amphiphilic substances (Kornhuber et al., 2010, 2011), tricyclic antidepressant analogs (Beckmann et al., 2014) and hydroxamic acid derivatives (Yang et al., 2020), have been under investigation as potential therapeutic agents. Evaluation of the tricyclic antidepressant amitriptyline in *ASAH1^−/−^* mice revealed reduced survival owing to toxicity effects (Beckmann et al., 2019), supporting the consideration of additional compound classes. Continued mechanistic studies and examination of pharmacological Smpd1 inhibitors in the *psap* KO and other sphingolipidosis models could shed further light on the feasibility of this approach for targeted sphingolipidosis therapy.

## MATERIALS AND METHODS

### Zebrafish

All zebrafish husbandry and experiment protocols were approved by and performed in accordance with the Institutional Animal Care and Use Committee at Massachusetts General Hospital or the University of Utah.

### Materials

Cas9 nuclease was purchased from New England Biolabs (M0386M). Proteinase K was purchased from Roche (03115828001). The Black Gold II staining kit was purchased from Millipore Sigma (AG105). 16% paraformaldehyde (PFA) was purchased from Electron Microscopy Sciences (15710). The JB-4 embedding kit was purchased from Electron Microscopy Sciences (14270-00) or Sigma-Aldrich (EM0100). The Embed 812 kit for electron microscopy was purchased from Electron Microscopy Sciences (14120). Embedding molds for adult fish (18646C-1) and block holders (15899) were purchased from Polysciences. Optimal Cutting Temperature (OCT) compound (25608-930) and cryomolds (25608-922) were purchased from VWR. Glass knives for sectioning of JB-4 blocks were cut from 8 mm glass strips purchased from Electron Microscopy Sciences (7890-08). Formalin was purchased from Sigma-Aldrich (F1635). Additional histology reagents were purchased from Electron Microscopy Sciences. MMF was purchased from Caymen Chemical (27813). RIPA buffer was purchased from Santa Cruz Biotechnology (sc-24948). PVDF membranes were purchased from Bio-Rad (162-0177; 162-0255). Antibodies were purchased from Cell Signaling Technology as follows: phospho-Akt (Ser473) (4060), phospho-Akt (Thr308) (13038, 4056), Akt (4691, 9272), phospho-p44/42 MAPK (Thr202/Tyr204) (4370), p44/42 MAPK (4695), phospho-p70S6K (9234), p70S6K (9202), phospho-4E-BP1 (2855), 4E-BP1 (9644), phospho-AMPKα (Thr172) (2535), AMPKα (5831), β-catenin (8480), β-actin (8457) and HRP-linked anti-rabbit IgG (7074); antibody validation was performed by Cell Signaling Technology based on their Hallmarks of Antibody Validation strategy, which was adapted from published work (Uhlen et al., 2016). The ECL Prime Western Blotting System was purchased from Millipore Sigma (GERPN2232). Glass Dounce homogenizers for lipid extraction were purchased from VWR (KT885300-0007). All solvents for lipidomics were LC–MS grade and purchased from VWR. PCR and cloning reagents were purchased from Promega. TruSeq Stranded Total RNA Library Prep with Ribo-Zero Gold was purchased from Illumina. RNA isolation and qRT-PCR reagents were purchased from QIAGEN and Thermo Fisher Scientific. Additional chemicals were purchased from Sigma-Aldrich and VWR. Oligonucleotides were synthesized at the Massachusetts General Hospital Center for Computational & Integrative Biology DNA Core, the University of Utah DNA Sequencing Core Facility, or Integrated DNA Technologies.

### Model generation

The *psap^+63/+63^* and *psap^−14/−14^* lines were generated and propagated following published protocol, using the TuAB strain (Zhang et al., 2019). The 20-nucleotide (N_20_) sequences were 5′-AGCTGTGGCAAGTCCCCTGT-3′, 5′-GGCAAGTCCCCTGTTGGGAA-3′, 5′-GGAACGGAGCAGTGTGCCCG-3′, 5′-GACATTCTGGCACCAGTAGG-3′ and 5′-GACAGCACCACAAAGGGAAG-3′. PCR parameters (Promega GoTaq) were: 95°C, 2 min; 36 cycles of 95°C for 30 s, 56°C for 30 s, and 72°C for 45 s; 72°C, 5 min. The forward primer was 5′-ACTTACTGGTCCTCCCATCTAA-3′ and the reverse primer was 5′-CACATTCTGTTGGCAGTGTTG-3′. The same primer sequences were used for DNA fragment analysis and Sanger sequencing. The forward primer was tagged with 6-FAM (Integrated DNA Technologies) at the 5′ end for DNA fragment analysis to enable fluorescence-based detection. Unmodified primers were used to generate the PCR product for sequencing. DNA fragment analysis and Sanger sequencing were performed at the Massachusetts General Hospital Center for Computational and Integrative Biology; the Genomics Core Facility, a part of the Health Sciences Cores at the University of Utah; or Genewiz. Data were analyzed in Geneious Prime. Additional DNA fragment analysis for the *psap^+63/+63^* and *psap^−14/−14^* lines was performed on the QIAxcel Advanced System (QIAGEN).

All homozygous *psap* KO zebrafish and WT siblings were obtained from group mating of heterozygous parents. Fin clipping was performed after graduation from nursery at 2-3 mpf, following which WT, heterozygous and homozygous populations were maintained in separate tanks at similar densities. Notably, we observed that earlier fin clipping correlated with longer survival {an example can be found in Fig. 7C (KO) and Fig. 7D [psap(KO), smpd1(WT)], where the former was maintained separately from WT siblings for compound treatment, while the latter was maintained with siblings until genotype determination at the end stage of disease}, suggesting that delayed separation of *psap* KO zebrafish from healthy siblings might have exacerbated pathologies in the former, possibly owing to increased competition for nutrients.

The *smpd1^−20/−20^* line was generated following same protocol as the *psap* KO model. N_20_ sequences were 5′-GGAGGAGGAAAACTATTGAC-3′, 5′-GACTCATACACAAACACTTG-3′, 5′-TGATTTCCATGCACAAACGG-3′, 5′-GTTACAGTGTATCCAGCGGT-3′ and 5′-GGGAAATCACGAGAGCACGC-3′. PCR parameters (Promega GoTaq) were 95°C, 2 min; 36 cycles of 95°C for 30 s, 51°C for 30 s, and 72°C for 45 s; 72°C, 5 min. The forward primer was 5′-GATATTGGGGGACGTATAGCAA-3′ and the reverse primer was 5′-ACAGCTTTACCGTATGGTCTCC-3′.

The *psap^+63/+^* and *smpd1^−20/−20^* lines (frozen sperm and live fish) are maintained at the University of Utah Centralized Zebrafish Animal Resource and available upon request.

### Tissue isolation

Adult zebrafish were euthanized by immersion in ice water. For brain isolation, the head was removed, the brain was rapidly excised from the ventral side, and either immediately placed into cold fixative or flash frozen in liquid nitrogen. The liver (from the ventral side) or the pair of optic nerves (containing the sections before and after the optic chiasm) was excised and immediately placed into cold fixative for TEM. Frozen brains were stored at −80°C and samples in fixative at 4°C prior to downstream processing.

### Lipid extraction

Lipid extraction was performed following previously published protocols (Zhang et al., 2019; Bligh and Dyer, 1959). Briefly, adult zebrafish brains were Dounce homogenized (30-40 times per sample) on ice in a mixture of 1.5:1.5:3.0 ml cold citric acid buffer (100 mM trisodium citrate, 1 M sodium chloride, pH 3.6):methanol:chloroform. The homogenized sample was vortexed for 15 s and centrifuged at 2000 ***g*** for 8 min to induce phase separation. The lipid-containing chloroform (bottom) layer was collected into a glass vial with a Pasteur pipette, transferred for a second time into a new glass vial to remove residual aqueous contamination, dried under nitrogen and stored at −80°C. All samples were analyzed within 2 weeks of processing.

### Lipidomics

Untargeted lipidomics was performed at the Harvard Center for Mass Spectrometry following a published protocol (Zhang et al., 2019). Data were acquired in MS/DD–MS^2^ (top5) mode on a Q Exactive Plus quadrupole-orbitrap mass spectrometer (Thermo Fisher Scientific) online with an Ultimate 3000 HPLC (Thermo Fisher Scientific). Positive and negative ionization modes were acquired separately. Raw files (.raw) were converted into .mzXML via msconvert (Chambers et al., 2012) and analyzed in R using the XCMS package (Smith et al., 2006). The identified lipid species were verified manually in using Xcalibur (Thermo Fisher Scientific), taking into account MS^1^ and MS^2^ data.

### Behavior tracking

Adult zebrafish were transferred into a 2.75-l fish tank (Aquatic Habitats) and allowed to acclimate for 10 min, after which the fish were filmed for 5 min from the side of the tank. Each zebrafish was filmed individually, alternating among WT, *psap^+63/+^* and *psap^+63/+63^* fish. All videos were processed in ActualTrack (ActualAnalytics) to yield mean swim speed data (Fig. 3D) and the time versus *x*/*y* positional data (Fig. 3C). The data shown in Fig. 3C were plotted in Python using the positional data generated by ActualTrack.

### Histology

Histology was performed using an established protocol (Sullivan-Brown et al., 2011) with the following modifications. Whole adult zebrafish were fixed in Dietrich's fixative (ZIRC Health Services Sample/Specimen Preparation) (30:10:2:58 ratio of 95% ethanol:formalin:glacial acetic acid:distilled water) for 1-2 weeks, followed by dehydration (20 min incubation step in 50% ethanol in PBS, followed by a 20 min incubation step in 75% ethanol in PBS and two 20 min incubation steps in 100% ethanol, all at room temperature), JB-4 infiltration and embedding. Embedding was performed overnight under static vacuum at 4°C. Then, 4-µm sections were collected on a Leica microtome (RM2125) with 8 mm glass blades and stained with H&E based on a previously published protocol (Sullivan-Brown et al., 2011).

### Whole-brain myelin staining

Following dissection, adult zebrafish brains were immediately placed in cold 4% PFA in PBS, and fixed for 1-2 days at 4°C. The fixed brains were washed twice with cold PBS, cryoprotected in 15% sucrose in PBS at 4°C for ∼24 h, washed twice with cold PBS, and cryoprotected in 30% sucrose in PBS at 4°C for ∼24 h. The cryoprotected brains were gently washed in a small volume of OCT at room temperature, placed into cryomolds with fresh OCT, frozen with liquid nitrogen and stored at −80°C. Each cryomold contained one WT brain next to one *psap^+63/+63^* brain frozen in the same orientation.

Frozen sections (20 µm) were collected for the entire brain on a cryotome at −16°C. Slides were stored at −80°C and stained within 2 weeks. Slides were post fixed in 10% formalin at room temperature for 1 h and rinsed briefly under tap water prior to staining. Myelin staining was performed using the Black Gold II staining kit, following the manufacturer's instructions; ∼1 h was typically needed for adequate stain development. All sections for each pair of WT and *psap^+63/+63^* brains were stained at the same time, and images were acquired for all stained sections to ensure consistent side-by-side comparisons between WT and *psap^+63/+63^* brain regions.

### Electron microscopy

TEM was performed at the University of Utah Electron Microscopy Core following established protocols (Yost et al., 2009). Briefly, optic nerves and livers were fixed in 2.5% glutaraldehyde and 1% paraformaldehyde in 0.1 M sodium cacodylate buffer with 2.4% sucrose and 8 mM calcium chloride (pH 7.40-7.45) at 4°C for a minimum of 24 h and up to 1 week. Fixed tissues were rinsed with 0.1 M sodium cacodylate buffer, post fixed with 2% osmium tetroxide in 0.1 M sodium cacodylate, dehydrated with a rising gradient of ethanol, and embedded in Embed 812. Next, 80 nm sections were cut using a diamond knife (Diatome) on an EMUC6 ultramicrotome (Leica) and placed on copper grids. Grids were contrasted with saturated aqueous uranyl acetate and Reynold's lead citrate sequentially. Images were acquired on an FEI Tecnai T-12 transmission electron microscope (Thermo Fisher Scientific) at 200 kV with a Gatan Ultrascan 1000 digital camera.

### RNA sequencing

RNA sequencing and data analyses were performed at the Huntsman Cancer Institute High-Throughput Genomics and Bioinformatic Analysis Shared Resource. RNA was isolated from adult zebrafish brains using the RNeasy Lipid Tissue Mini kit (QIAGEN, 74804; four biological replicates each of WT and *psap^+63/+63^*; each replicate contained RNA isolated from three pooled brains). cDNA library was prepared using the TruSeq Stranded Total RNA Library Prep Ribo-Zero Gold kit (Illumina). All samples were pooled for sequencing on the Illumina NovaSeq 6000 Sequencing System (2×150 bp, 300 million reads total).

For data analysis, the zebrafish Zv10 genome and gene feature files were downloaded from Ensembl release 94 (Zerbino et al., 2017), and the reference database was created using STAR version 2.6.1b with splice junctions optimized for 150 base pair reads (Dobin et al., 2013). Optical duplicates were removed from the paired end FASTQ files using clumpify v38.34 of BBMap, and reads were trimmed of adapters using cutadapt 1.16 (Martin, 2011). The trimmed reads were aligned to the reference database using STAR in two pass mode to output a BAM file sorted by coordinates. Mapped reads were assigned to annotated genes using featureCounts version 1.6.3 (Liao et al., 2014). The output files from cutadapt, FastQC, Picard CollectRnaSeqMetrics, STAR and featureCounts were summarized using MultiQC to check for any sample outliers (Ewels et al., 2016). Differentially expressed genes were identified using a 5% false discovery rate with DESeq2 version 1.22.2 (Love et al., 2014). Pathways were analyzed using the fast gene set enrichment (fgsea) package (Korotkevich et al., 2021 preprint).

### qRT-PCR

Zebrafish brain and larva samples were processed using the RNeasy Lipid Tissue Mini kit (QIAGEN, 74804) and RNeasy Tissue Mini kit (QIAGEN, 74104), respectively. Isolated RNA (1 μg input per sample) was reverse transcribed via the QuantiTect Reverse Transcription kit (QIAGEN, 205313), and qRT-PCR was performed with PowerUp SYBR Green Master Mix (Thermo Fisher Scientific, A25742). 500 nM of each primer and 18.75 ng cDNA per sample was used to carry out a 15-μl reaction on the Applied Biosystems 7500 Fast Real-Time PCR System (Thermo Fisher Scientific). PCR parameters were as follows: 50°C, 2 min; 95°C, 2 min; 40 cycles of 95°C for 15 s, 60°C for 15 s, and 72°C for 1 min. Melt curve analysis was performed at the end of each sample run using the default continuous option in 7500 Software v.2.3 (Thermo Fisher Scientific). Data were analyzed and reported as relative expression using the ΔΔCt method. Primer sequences are listed in Table S6.

### Protein expression analysis

All samples were lysed in RIPA buffer (Santa Cruz Biotechnology, sc-24948) prior to gel electrophoresis and western blotting. Approximately 30-40 µg protein was loaded per lane. β-actin was used as a loading control. With the exceptions of the phospho-p44/42 MAPK and p44/42 MAPK antibodies, which could not be stripped efficiently due to tight binding, each phosphorylated protein and its unphosphorylated counterpart were probed on the same membrane using stripping buffer (Abcam; 20 ml 10% SDS; 12.5 ml 1.5 M Tris-HCl, pH 6.8; 67.5 ml water; 0.8 ml β-mercaptoethanol) to remove the phosphoprotein prior to reblotting. All antibodies were diluted in blocking buffer (Rockland, MB-070). The dilutions used were 1:500-1:1000 for p-p70S6K, 1:3000 for β-actin and 1:1000 for all additional antibodies. PVDF membranes were incubated with ECL solution (Millipore Sigma, GERPN2232) following the manufacturer's instructions for 1-5 min and imaged on a Fluor Chem M imager (Bio-Techne).

### MMF treatment of juvenile and adult zebrafish

WT, untreated *psap^+63/+63^*, and MMF-treated *psap^+63/+63^* fish were raised in system water at a density of ∼7 fish/l for juvenile fish and ∼9 fish/l for adult fish. MMF-containing system water was prepared at 100 µM by directly dissolving the compound in the system water without additional carrier solvent. All fish were maintained in system water without circulation and 100% of the water (with or without freshly prepared compound) was exchanged daily. Juvenile fish were treated from 14 to 33 dpf, and adult fish from 3.5 mpf until the time of euthanasia owing to severe pathology, defined as difficulty maintaining neutral buoyancy and/or cachexia. Juvenile fish were fed once daily with brine shrimp, and adult fish twice daily (brine shrimp in the morning, dried fish food in the afternoon) on weekdays and once daily (dried fish food at twice the amount as weekday afternoon feedings) on weekends.

### *smpd1* mutagenesis in *psap* KO zebrafish

*Psap^+63/+^* zebrafish were crossed with *smpd1^−20/−20^* zebrafish, and the offspring were genotyped to obtain the doubly heterozygous (*psap^+63/+^, smpd1^−20/+^*) population, which was incrossed to yield a population carrying a mixture of WT, heterozygous and homozygous *psap* and *smpd1* alleles. Embryos from the doubly heterozygous incross were raised to adulthood at a density of ∼10 fish/l. Starting from 3 mpf, these fish were monitored every two days and fish reaching the end stage of disease (defined as difficulty maintaining neutral buoyancy and/or cachexia) were euthanized and genotyped using the tail fin, until no fish with visible phenotypes remained in the studied population. All euthanized fish were homozygous at the *psap* locus (103 fish out of a total of ∼440 fish, or ∼23%). An additional 82 fish without observable phenotypes were also genotyped at the end of the experiment to reveal no *psap^−/−^* fish within this population. The recorded number of fish, time of euthanasia and associated genotypes were used to generate Fig. 7D.

### Statistics

Differential expression analysis for RNA sequencing was performed via the DESeq2 function operated in R; a detailed description of the statistical analysis package (Love et al., 2014) and R vignettes have been previously published. Statistical testing for survival analysis (Fig. 7C,D) was performed using the log-rank (Mantel–Cox) test (Prism 9). All additional statistical testing was performed using Student's *t*-test (Microsoft Excel, unpaired, two-tailed, two-sample equal variance option was used based on identical sample type and similar sample sizes between WT and KO cohorts). A mixture of male and female zebrafish was used in all studies involving the late juvenile-adult stage, and all samples were processed in an alternating sequence of WT and KO. The sample size was the maximum number of zebrafish that could be allocated to each experiment, as the effect size was not known prior to experiment. With the exception of the *smpd1* knockout rescue, the genotype of the fish was not hidden. Fish were only excluded from analysis in instances (∼1% of all PCR reactions) in which genotyping yielded inconclusive data.

## Supplementary Material

10.1242/dmm.049995_sup1 Supplementary information
