# Supplementary material for: A zebrafish model of combined saposin deficiency identifies acid sphingomyelinase as a potential therapeutic target
Source: Dis Model Mech. 2023 Jun 27;16(7):dmm049995. doi: 10.1242/dmm.049995 (PMC10320721; doi:10.1242/dmm.049995)
Supplement: Supplementary information [file dmm-16-049995-s1.pdf]

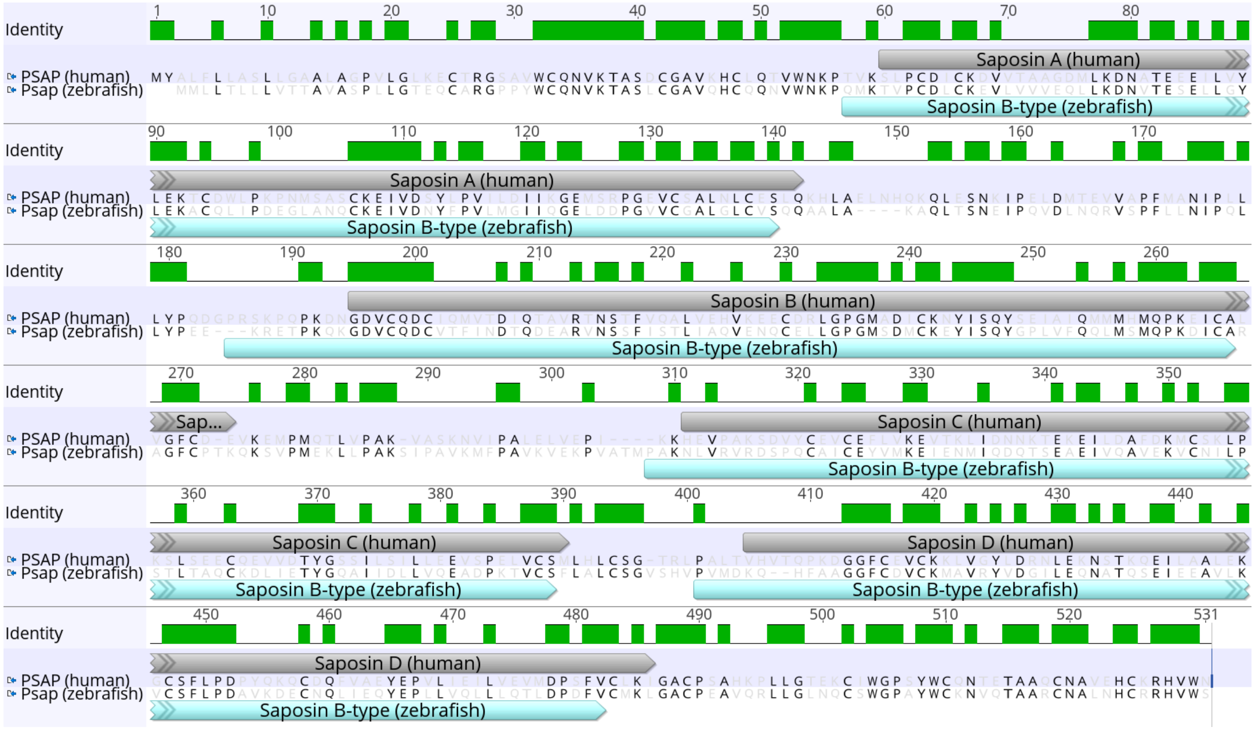

Fig. S1, related to Figure 1. Amino acid identities between human and zebrafish saposin proteins. (A) Amino acid sequence alignment of human (UniProt: P07602) and zebrafish prosaposin (UniProt: B8JI17). Green highlights indicate conserved residue(s). Human saposin A-D domains and the corresponding zebrafish saposin-like domains are annotated in the figure. Saposin sequence and domain information was from UniProt.[1] Alignment was performed in Geneious Prime.

Disease Models & Mechanisms • Supplementary information

A

psap$^{+63/+63}$

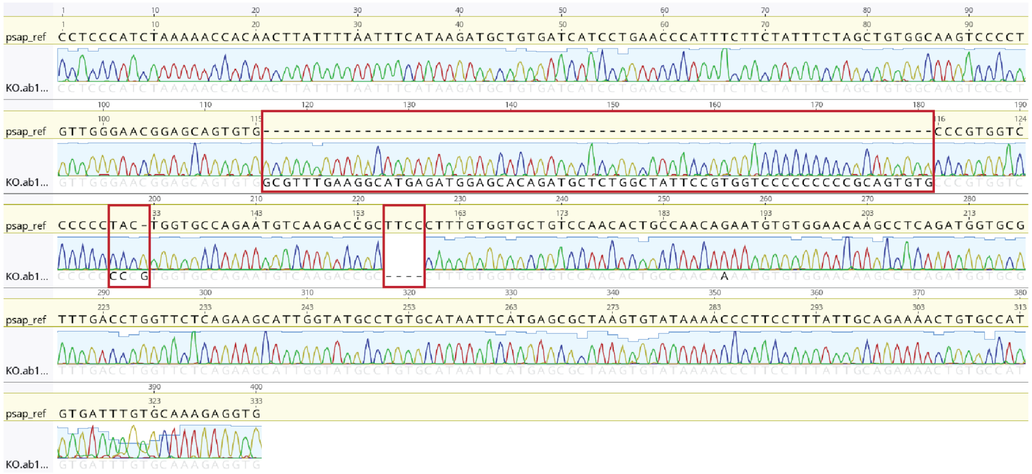

B

psap$^{-14/-14}$

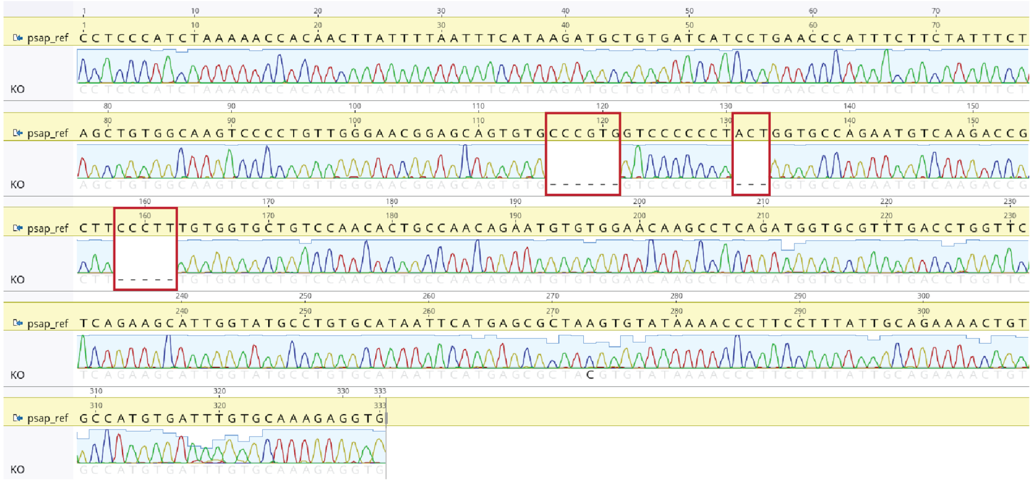

C

psap$^{+/+}$

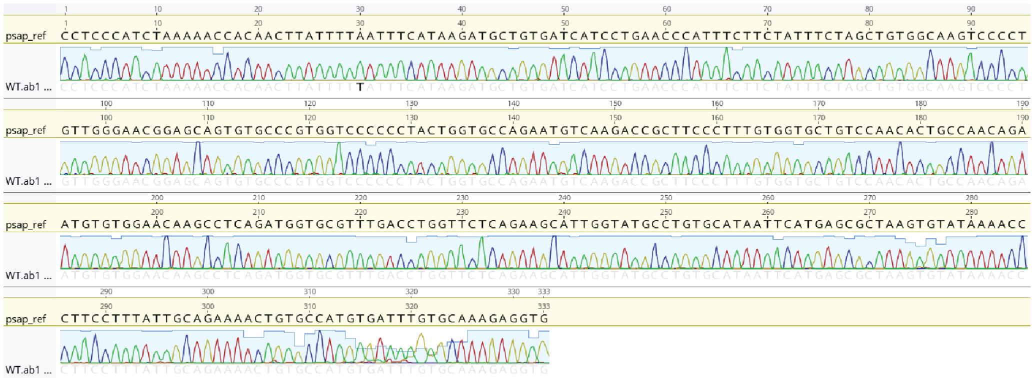

Fig. S2, related to Figure 1. Sanger sequencing of WT and *psap* zebrafish. **(A)** Alignment of Sanger sequencing result of *psap* PCR product from *psap*$^{+63/+63}$ zebrafish against reference sequence. Guides target exon 2 of *psap* gene. Boxed regions indicate mutations. **(B)** Alignment of Sanger sequencing result of *psap* PCR product from *psap*$^{-14/-14}$ zebrafish against reference sequence. Boxed regions indicate mutations. **(C)** Alignment of Sanger sequencing result of *psap* PCR product from WT zebrafish against reference sequence. Reference sequence is the WT *psap* PCR product based on zebrafish genome assembly GRCz11.[2] All alignments are performed in Geneious Prime.

Disease Models & Mechanisms • Supplementary information

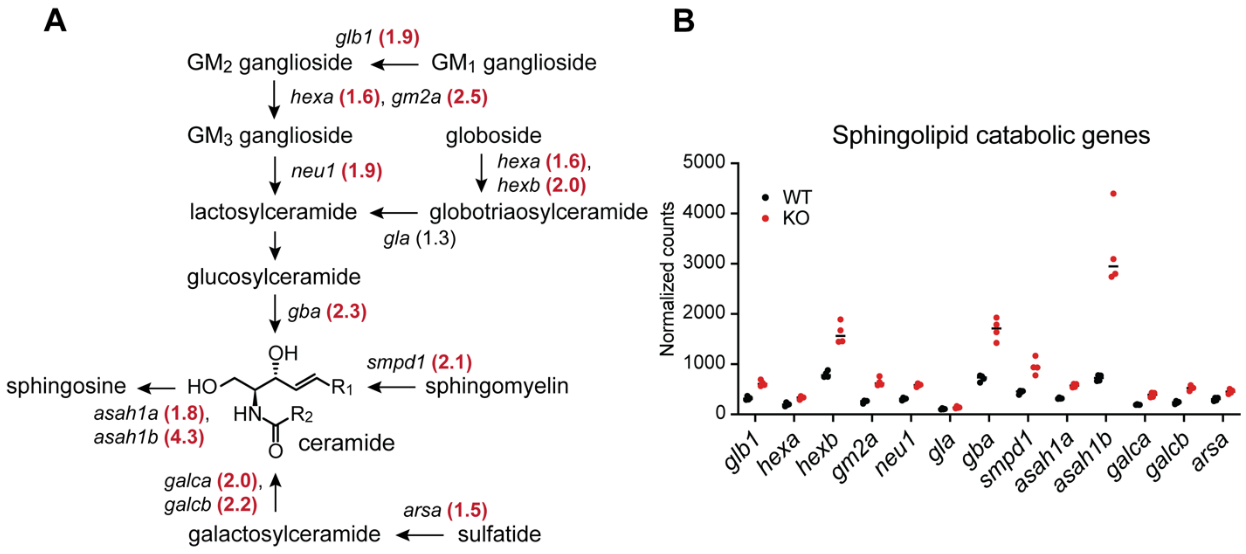

Fig. S3, related to Figure 1. Effect of combined saposin deficiency on genes of lysosomal sphingolipid catabolism. (A) The sphingolipid metabolic pathway, number in parenthesis indicates *psap*/WT fold change for the associated gene from RNA sequencing (**Table S3**) of 4 mpf WT (n = 4) and *psap*$^{+63/+63}$ (n = 4) zebrafish brains. **(B)** Relative transcript levels of the genes of lysosomal sphingolipid catabolism indicated in **(A)**. Adjusted p-values (**Table S3**) < 0.001 for all genes.

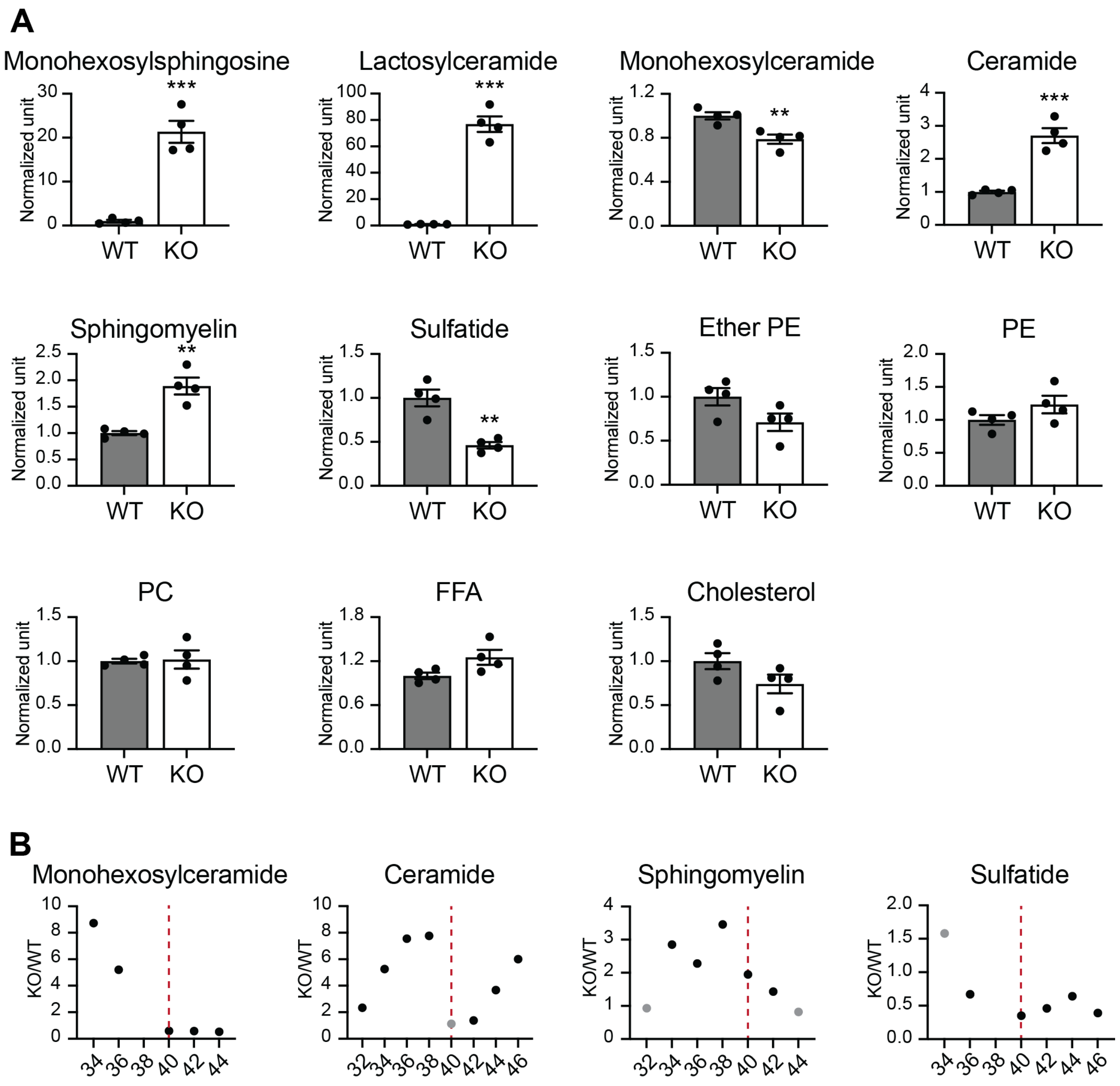

Fig. S4, related to Figure 2. Altered lipid metabolism in the *psap* zebrafish. (A) Relative levels of major lipid families in 4 mpf WT (n = 4) and *psap*$^{-14/-14}$ (n = 4) brains. **(B)** KO/WT fold changes vs. number of carbons in the ceramide core of the lipid species for selected sphingolipid families. Two-tailed Student's t-test; **p < 0.01, ***p < 0.001; mean ± SEM.

Disease Models & Mechanisms • Supplementary information

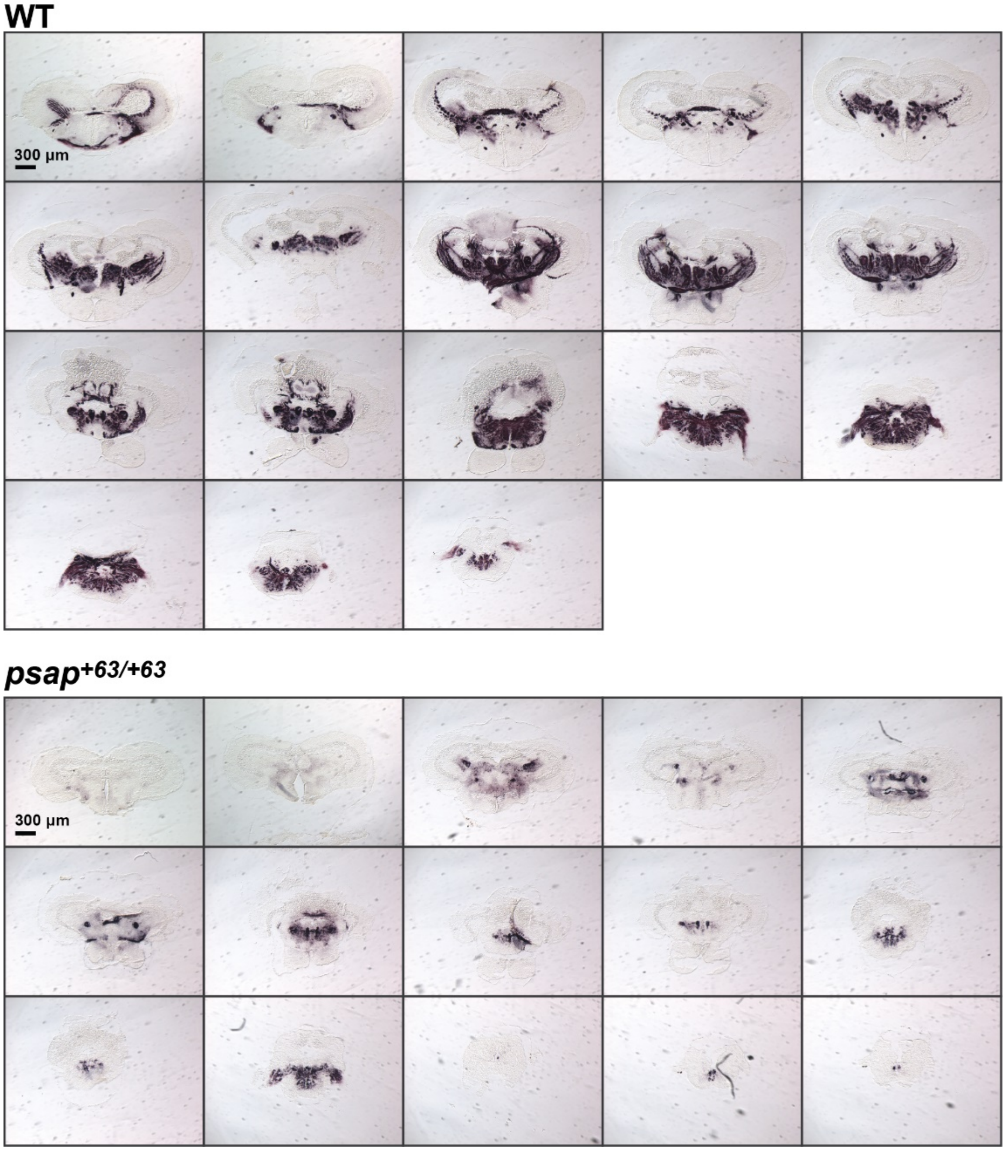

Fig. S5, related to Figure 3. Myelination across serial WT and *psap*$^{+63/+63}$ brain sections. Black-Gold II staining of serial frozen brain sections (anterior to posterior), demonstrating severe myelin loss across most brain regions.

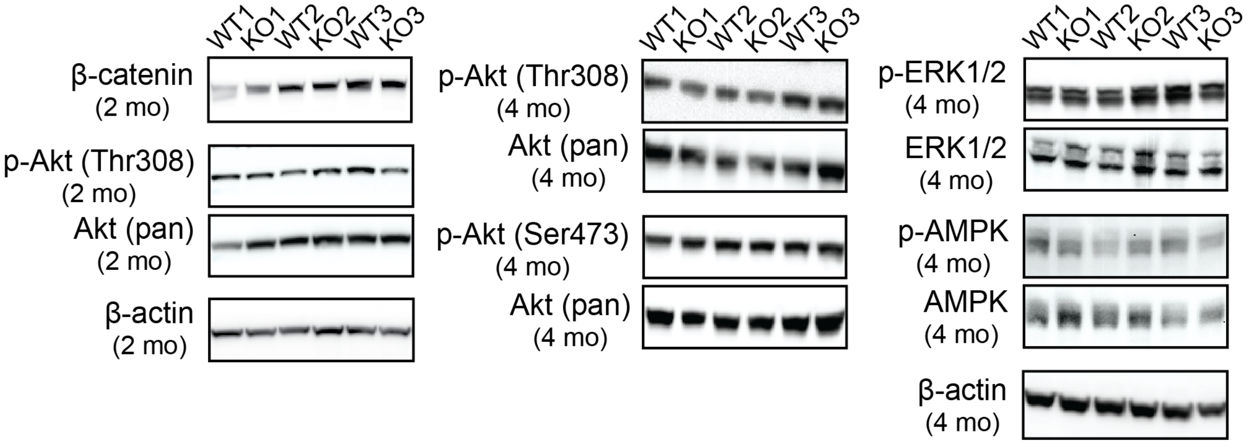

Fig. S6, related to Figure 5. Western blot of additional promyelinating pathways in *psap*$^{+63/+63}$ and WT sibling zebrafish brains at 2 and 4 mpf.

Disease Models & Mechanisms • Supplementary information

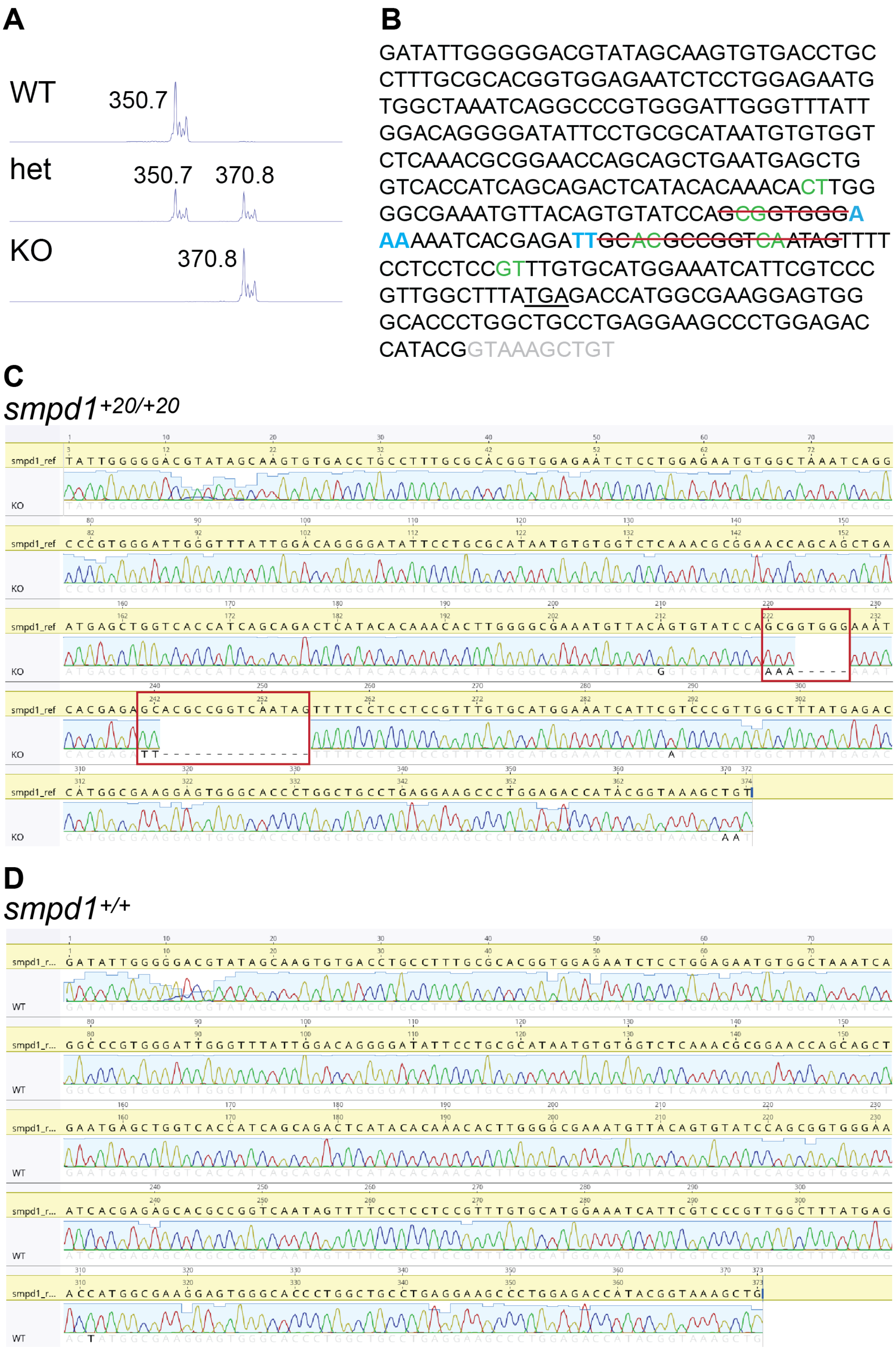

Disease Models & Mechanisms • Supplementary information

Fig. S7, related to Figure 7. DNA fragment analysis and Sanger sequencing of a zebrafish model of Niemann-Pick Disease Type A, B. (A) DNA fragment analyses of *smpd1* PCR fragments from WT and mutant zebrafish, demonstrating the presence of a net 20-bp deletion in the mutant sample. **(B)** Sanger sequencing of DNA fragment from **Fig. S6A**. Exon regions are in black and introns are in gray. Insertions are in blue and deletions are crossed out. Cas9 cut sites are in green. The premature stop codon is underlined. All CRISPR-Cas9 guides target exon 2 (out of 6) of zebrafish *smpd1*. **(C)** Alignment of Sanger sequencing result of *smpd1* PCR product from *smpd1*$^{-20/-20}$ zebrafish against reference sequence (WT *smpd1* PCR product based on zebrafish genome assembly GRCz11[2]). Boxed regions indicate mutations. **(D)** Alignment of Sanger sequencing result of *smpd1* PCR product from WT sibling zebrafish against reference sequence (WT *psap* PCR product based on zebrafish genome assembly GRCz11[2]).

Disease Models & Mechanisms • Supplementary information

Table S1, related to Figure 2. List of validated lipid species by sphingolipid class from lipidomics analysis of 4 mpf *psap*$^{+63/+63}$ and WT sibling brains.

Click here to download Table S1

Table S2, related to Figure 2. List of validated lipid species by sphingolipid class from lipidomics analysis of 4 mpf *psap*$^{-14/-14}$ and WT sibling brains.

Click here to download Table S2

Table S3, related to Figure 5. Differential analysis table of all annotated genes from brain transcriptomics of 4 mpf *psap*$^{+63/+63}$ zebrafish and WT siblings. The full list of genes with fold change and adjusted p-values is in the psap_vs_WT (highlighted) tab.

Click here to download Table S3

Table S4, related to Figure 5. Gene set enrichment analyses of zebrafish brain transcriptomics data. **Fig. 5B** was created based on data in the Hallmark tab.

Click here to download Table S4

Table S5, related to Figure 5. Gene list for hierarchical clustering analysis (**Fig. 5C**). Gene list was manually curated by finding the zebrafish orthologs of the common brain cell type markers listed in the Bio-Techne Neural Cell Markers reference[47].

Click here to download Table S5

Table S6. Primer sequences for qRT-PCR

Gene	Forward primer	Reverse primer
mbpa	CGGAGGAGACAAGAAGAGAAAG	TCCTGAAGAAATGCACGACA
mbpb	AACCATTGTTTCTCCCTCTACC	TTAGGGTGCCTTGACTCTCT
stat2	CTCTCGTTGAGTGTGACAGAAG	GAAGAGAAGAGGTCTCCAGAGT
stat3	GGTGTGGCTGGACAACATTA	CTCAGGATTGCTCTCTCTCTTTC
jak1	CCAGAAACAAGGCTCACATAAAC	CAGGTCTCTGTGGATGTAGTTG
socs1a	GGAAGGGAATCAAGGCATAGAA	CTGGATCTTTGGGATTGGAAGA
socs1b	TCCCACCAACACACTTTCAC	AGCTTCGTCCACATCCAAAG
nfkb1	CTCCAGCATCAGTTTCCCTAAT	CGCTCCTCCAACACTTTACTC
nfkb2	TGAGGCTGCTGAGAGATAGT	TGCCATCTCCTCTCCAATAGA
nfkbiaa	ATGGTGGAAAGACTCCTGAAAG	CTGAGTGAGGACTGAGAAACAC
nfkbiab	CACGTATCTACATCTCGCCATT	GAAAGGGTCTCTTCGGGATAAC
il1b	GGAGACCATTAAAGCTGGAGAT	GACGGACTCGAAGGTGTTTAT
tnfb	GGTCAGAAACCCAACAGAGAA	CTTTGTGCTCTGAGCGATCT
olig2	AAATAGCCACTCTGCTGCTC	ACAACTGGACGGATGGAAAC
Pdgfra	GTGGGTGAGACTATCACTGTTG	ACAGTCTTTGTTCCACGATTAG
cspg4	GGCAGATTACAGGCTAGGATTG	GAGACACATGGTGGTCAAGAAG
cnp	AGACGCTGGATACATTGGAAG	GGCAGTGGGTTCTGGAAATA
myrf	TGGGAGCGTTAGTGATGTTG	AGACAGGAGGATGGACAGAA
plp1a	GCCCGTGACAATCAAGACTA	CTTCAGCCAGCAGGACTATAC
plp1b	GCCTCCTTCTTCTTCCTCTATG	GAACTGTGTGCTCCTGAACT
mag	ACTTCCCAAACACCACCTAC	CCTCCATCACCTCTTGAGATAC
cd45	CTGATGAGAGCCGACTGTTTAT	CGCCTCCTTGATGGTGTAAT
cd68	AACACAACCACTCCAATCCC	TGCAAAGTCAGCCAGAATACA
gfap	CAGATGCTGAAGGAGGAGATG	CTTCCTGTAGGTGGCGATTT
rbfox3a	TGGTACAGACAGACGGATCT	GGGAATGTTGGAGACGTGTAA
rbfox3b	GGGCTTTGGCTTTGTAACTTT	TCCTTCTACGATTGTACCGTTTAG
18s	TCGCTAGTTGGCATCGTTTATG	CGGAGGTTCGAAGACGATCA

REFERENCES

1 UniProt Consortium. UniProt: a worldwide hub of protein knowledge. *Nucleic Acids Research* **47**, D506-D515 (2018). https://doi.org/10.1093/nar/gky1049

2 Frankish, A. *et al.* Ensembl 2018. *Nucleic Acids Research* **46**, D754-D761 (2017). https://doi.org/10.1093/nar/gkx1098

Disease Models & Mechanisms • Supplementary information
